# Supplementary material for: Symptoms and treatment response to florensocatib and inhaled tobramycin in bronchiectasis: Post hoc analysis of two randomized trials
Source: Cell Rep Med. 2026 Aug 13;7(9):102995. doi: 10.1016/j.xcrm.2026.102995 (PMC13589483; doi:10.1016/j.xcrm.2026.102995)
Supplement: Supplementary file 1 [file mmc1.pdf]

Cell Reports Medicine, Volume 7

Supplemental information

Symptoms and treatment response to florensocatib and inhaled tobramycin in bronchiectasis: Post hoc analysis of two randomized trials

Yong-Hua Gao (高永华), Xue-Shan Li (李雪杉), Xiao-Li Sun (孙晓丽), Fang-Qiong Li (李芳琼), Lu-Yin Sun (孙露银), Han-Mo Liu (刘翰墨), and Wei-Jie Guan (关伟杰)

Supplemental information

Symptoms and Treatment Response to DPP-1 Inhibitor Florensocatib and Inhaled Tobramycin in Adults with Bronchiectasis: *Post-hoc* Analysis of Two Multicenter Randomized, Placebo-Controlled Trials

Yong-Hua Gao, Xue-Shan Li, Xiao-Li Sun, Fang-Qiong Li, Lu-Yin Sun, Han-Mo Liu, Wei-Jie Guan.

Supplementary tables

Table S1. Baseline symptom burden assessed by QoL-B-RSS of patients with bronchiectasis in SAVE-BE and TORNASOL trial. Related to Table 1 and Table 2.

**Symptom burden groups**	**SAVE-BE trial**				**TORNASOL trial**			
	**Total (N=224)**	**Florensocatib (n=149)**	**Placebo (n=75)**	***P*-value**	**Total (N=357)**	**Tobramycin (n=178)**	**Placebo (n=179)**	***P*-value**
High-symptom group, n (%)	107 (47.8%)	71 (47.7%)	36 (48.0%)	0.961	152 (42.6%)	84 (47.2%)	68 (38.0%)	0.079
Low-symptom group, n (%)	117 (52.2%)	78 (52.3%)	39 (52.0%)		205 (57.4%)	94 (52.8%)	111 (62.0%)	

All values are n (%). High-symptom burden: QoL-B-RSS<60; low-symptom burden: QoL-B-RSS≥60. Definition of abbreviations: QoL-B-RSS=Quality-of-Life Bronchiectasis Respiratory Symptoms Scale.

Table S2. Sputum characteristics, lung function and respiratory symptoms at week 24 stratified by different symptom burden subgroups in the SAVE-BE trial. Related to Figure 1 and Figure 2.

Endpoints and subgroups	Florensocatib		Placebo		Mean difference versus placebo (95%CI)	*P*-value
	No.	LSM±SE	No.	LSM±SE		
**24-hour sputum weight, g**						
All patients in SAVE-BE trial	132	-8.13±1.83	64	0.29±2.50	-8.43 (-14.00 to -2.85)	0.0032
High-symptom group	60	-14.20±3.65	31	-3.32±4.60	-10.89 (-21.46 to -0.31)	0.0437
Low-symptom group	72	-2.89±1.61	33	2.88±2.34	-5.77 (-10.93 to -0.62)	0.0286
**Sputum purulence score, points**						
All patients in SAVE-BE trial	132	-0.70±0.17	64	0.10±0.23	-0.80 (-1.28 to -0.26)	0.0031
High-symptom group	60	-0.70±0.25	31	0.00±0.31	-0.70 (-1.38 to 0.04)	0.0653
Low-symptom group	72	-0.60±0.23	33	0.30±0.33	-0.90 (-1.64 to -0.20)	0.0133
**FEV_1 percentage predicted, %**						
All patients in SAVE-BE trial	133	-1.71±0.68	63	-2.43±0.93	0.72 (-1.33 to 2.77)	0.4896
High-symptom group	61	-1.81±1.03	31	-1.38±1.28	-0.43 (-3.37 to 2.51)	0.7727
Low-symptom group	72	-1.70±0.93	32	-3.46±1.37	1.76 (-1.23 to 4.74)	0.2457
**QoL-B-RSS, points**						
All patients in SAVE-BE trial	133	5.73±1.26	64	3.90±1.68	1.83 (-1.93 to 5.60)	0.3380
High-symptom group	61	8.55±2.13	31	11.30±2.59	-2.75 (-8.91 to 3.41)	0.3770
Low-symptom group	72	2.20±1.48	33	-2.33±2.13	4.53 (-0.19 to 9.25)	0.0598

The LSE±SE of different treatment represents the changes from baseline to week 24. All estimates have been adjusted with the baseline levels and the exacerbation frequency within the previous year (cut-off: 3 events). High-symptom group: QoL-B-RSS<60; low-symptom group: QoL-B-RSS≥60. QoL-B-RSS: Quality-of-Life of Bronchiectasis Respiratory Symptom Scale; FEV_1: forced expiratory volume in one second; LSM: least-square mean; SE: standard error; CI: confidence interval.

Table S3. Mean changes from baseline in individual respiratory symptoms of QoL-B-RSS in patients receiving florensocatib versus placebo in the SAVE-BE trial. Related to Figure 1 and Figure 2.

Items	Visits	Florensocatib		Placebo (Mean±SE)		Mean Difference vs placebo (95%CI)	*P*-value
		No.	Mean±SE	No.	Mean±SE		
Congestion							
	Week 4	144	0.19±0.98	72	0.24±0.97	-0.06 (-0.28- 0.16)	0.6071
	Week 12	137	0.32±1.13	66	0.24±1.19	0.10 (-0.15- 0.36)	0.4358
	Week 20	133	0.32±1.00	64	0.28±1.13	0.07 (-0.17- 0.31)	0.5602
	Week 24	133	0.34±0.99	64	0.33±1.04	0.06 (-0.18- 0.30)	0.6061
Daily cough							
	Week 4	144	0.05±0.80	72	0.08±0.92	0.00 (-0.21- 0.21)	0.9998
	Week 12	137	0.30±0.81	66	0.24±0.79	0.11 (-0.10- 0.32)	0.3090
	Week 20	133	0.31±0.83	64	0.30±0.85	0.08 (-0.12- 0.29)	0.4265
	Week 24	133	0.28±0.76	64	0.20±0.78	0.14 (-0.06- 0.34)	0.1594
Sputum production							
	Week 4	144	0.09±0.98	72	0.32±0.98	-0.16 (-0.41- 0.08)	0.1935
	Week 12	137	0.28±0.95	66	0.30±0.91	0.07 (-0.19- 0.32)	0.6016
	Week 20	133	0.27±0.90	64	0.36±0.95	-0.01 (-0.25- 0.23)	0.9562
	Week 24	133	0.34±0.89	64	0.31±0.83	0.09 (-0.14- 0.33)	0.4299
Sputum purulence							
	Week 4	144	-0.02±0.72	72	-0.06±0.65	0.02 (-0.16- 0.20)	0.8255
	Week 12	137	0.01±0.69	66	-0.11±0.73	0.09 (-0.10- 0.27)	0.3628
	Week 20	133	0.09±0.70	64	-0.16±0.70	0.24 (0.05- 0.44)	0.0140
	Week 24	133	-0.02±0.74	64	-0.13±0.71	0.09 (-0.11- 0.29)	0.3842
Breathlessness on daily activity							
	Week 4	144	0.10±0.81	72	0.13±0.77	0.03 (-0.17- 0.23)	0.7539
	Week 12	137	0.18±0.85	66	0.14±0.93	0.13 (-0.09- 0.34)	0.2533
	Week 20	133	0.24±0.87	64	0.23±0.87	0.09 (-0.13- 0.30)	0.4240
	Week 24	133	0.21±0.90	64	0.17±0.77	0.11 (-0.11- 0.33)	0.3210
Wheeze							
	Week 4	144	0.10±0.72	72	0.15±0.87	0.03 (-0.16- 0.22)	0.7608
	Week 12	137	0.23±0.83	66	0.18±0.98	0.15 (-0.05- 0.35)	0.1394
	Week 20	133	0.22±0.71	64	0.23±0.83	0.07 (-0.12- 0.26)	0.4556

	Week 24	133	0.20±0.69	64	0.28±0.77	0.01 (-0.16- 0.18)	0.9036
Chest pain							
	Week 4	144	0.01±0.55	72	0.03±0.73	-0.01 (-0.16- 0.15)	0.9240
	Week 12	137	0.17±0.68	66	0.15±0.79	0.03 (-0.13- 0.20)	0.6997
	Week 20	133	0.19±0.59	64	0.16±0.72	0.05 (-0.10- 0.19)	0.5270
	Week 24	133	0.15±0.63	64	0.08±0.67	0.09 (-0.06- 0.23)	0.2525
Breathlessness on talking							
	Week 4	144	0.02±0.61	72	0.17±0.80	-0.05 (-0.23- 0.13)	0.5782
	Week 12	137	0.08±0.63	66	0.17±0.81	0.01 (-0.17- 0.20)	0.8909
	Week 20	133	0.02±0.63	64	0.19±0.83	-0.04 (-0.23- 0.15)	0.6932
	Week 24	133	0.02±0.72	64	0.16±0.70	0.00 (-0.19- 0.19)	0.9986
Nocturnal cough							
	Week 4	144	0.10±0.62	72	0.07±0.66	0.11 (-0.05- 0.26)	0.1728
	Week 12	137	0.20±0.63	66	0.14±0.78	0.14 (-0.03- 0.30)	0.1125
	Week 20	133	0.20±0.67	64	0.14±0.83	0.17 (-0.01- 0.35)	0.0587
	Week 24	133	0.08±0.62	64	0.11±0.80	0.07 (-0.10- 0.24)	0.4358

Mean changes from baseline in individual QoL-B-RSS symptom domains between florensocatib and placebo. Abbreviations: QoL-B-RSS=Quality-of-Life Bronchiectasis Respiratory Symptoms Scale; SE: standard error; CI: confidence interval.

Table S4. Mean changes from baseline in individual respiratory symptoms of QoL-B-RSS in patients with high symptom burden receiving florensocatib versus placebo in the SAVE-BE trial. Related to Figure 1 and Figure 2.

Items	Visits	Florensocatib		Placebo (Mean±SE)		Mean Difference vs placebo (95%CI)	*P*-value
		No.	Mean±SE	No.	Mean±SE		
Congestion							
	Week 4	68	0.50±0.87	35	0.57±0.92	-0.06 (-0.38- 0.26)	0.7199
	Week 12	64	0.67±1.05	32	0.75±1.16	-0.04 (-0.44- 0.37)	0.8624
	Week 20	61	0.61±1.04	30	0.87±1.01	-0.16 (-0.57- 0.24)	0.4318
	Week 24	61	0.62±0.93	31	0.81±1.01	-0.13 (-0.50- 0.25)	0.5082
Daily cough							
	Week 4	68	0.18±0.90	35	0.23±1.03	0.00 (-0.33- 0.32)	0.9890
	Week 12	64	0.48±0.94	32	0.50±0.88	0.03 (-0.34- 0.39)	0.8876
	Week 20	61	0.49±0.89	30	0.63±0.76	-0.08 (-0.42- 0.26)	0.6467
	Week 24	61	0.39±0.86	31	0.45±0.81	-0.01 (-0.35- 0.32)	0.9324
Sputum production							
	Week 4	68	0.25±1.03	35	0.63±0.94	-0.23 (-0.61- 0.15)	0.2338
	Week 12	64	0.45±0.89	32	0.66±0.87	-0.12 (-0.50- 0.26)	0.5417
	Week 20	61	0.41±0.90	30	0.70±0.95	-0.13 (-0.52- 0.25)	0.5005
	Week 24	61	0.48±0.83	31	0.61±0.92	-0.03 (-0.39- 0.33)	0.8721
Sputum purulence							
	Week 4	68	0.02±0.75	35	0.06±0.59	-0.01 (-0.27- 0.24)	0.9097
	Week 12	64	0.00±0.74	32	0.00±0.72	0.03 (-0.24- 0.29)	0.8451
	Week 20	61	0.14±0.63	30	-0.10±0.66	0.29 (0.04- 0.55)	0.0239
	Week 24	61	0.05±0.65	31	-0.03±0.61	0.09 (-0.18- 0.35)	0.5136
Breathlessness on daily activity							
	Week 4	68	0.25±0.92	35	0.09±0.92	-0.01 (-0.35- 0.32)	0.9315
	Week 12	64	0.36±0.82	32	0.25±0.95	-0.02 (-0.35- 0.32)	0.9249
	Week 20	61	0.39±0.90	30	0.30±0.84	-0.03 (-0.38- 0.31)	0.8414
	Week 24	61	0.36±0.91	31	0.26±0.73	-0.03 (-0.36- 0.31)	0.8667
Wheeze							
	Week 4	68	0.22±0.91	35	0.26±1.01	0.01 (-0.32- 0.34)	0.9367
	Week 12	64	0.59±0.92	32	0.19±0.93	0.31 (-0.02- 0.64)	0.0628
	Week 20	61	0.39±0.88	30	0.33±0.99	-0.07 (-0.41- 0.27)	0.6808

	Week 24	61	0.43±0.83	31	0.29±0.82	-0.11 (-0.40- 0.19)	0.4766
Chest pain							
	Week 4	68	0.04±0.53	35	0.09±0.92	-0.02 (-0.26- 0.22)	0.8707
	Week 12	64	0.28±0.79	32	0.25±0.95	0.07 (-0.20- 0.35)	0.5934
	Week 20	61	0.28±0.71	30	0.30±0.84	0.01 (-0.23- 0.26)	0.9168
	Week 24	61	0.26±0.73	31	0.26±0.73	0.02 (-0.22- 0.26)	0.8693
Breathlessness on talking							
	Week 4	68	0.01±0.82	35	0.26±1.01	-0.11 (-0.44- 0.22)	0.5162
	Week 12	64	0.16±0.78	32	0.19±0.93	0.06 (-0.26- 0.38)	0.7115
	Week 20	61	0.11±0.75	30	0.33±0.99	-0.07 (-0.40- 0.27)	0.6807
	Week 24	61	0.11±0.80	31	0.29±0.82	-0.03 (-0.35- 0.29)	0.8390
Nocturnal cough							
	Week 4	68	0.19±0.65	35	0.29±0.71	0.01 (-0.23- 0.26)	0.9138
	Week 12	64	0.31±0.73	32	0.25±0.95	0.16 (-0.13- 0.46)	0.2713
	Week 20	61	0.39±0.71	30	0.33±0.92	0.29 (0.01- 0.57)	0.0453
	Week 24	61	0.20±0.60	31	0.23±0.88	0.15 (-0.10- 0.39)	0.2432

Mean changes from baseline in individual QoL-B-RSS symptom domains between florensocatib and placebo in patient with high symptom burden (QoL-B-RSS<60).

Abbreviations: QoL-B-RSS=Quality-of-Life Bronchiectasis Respiratory Symptoms Scale; SE: standard error; CI: confidence interval.

Table S5. Mean changes from baseline in individual respiratory symptoms of QoL-B-RSS in patients with low symptom burden receiving florensocatib versus placebo in the SAVE-BE trial. Related to Figure 1 and Figure 2.

Items	Visits	Florensocatib		Placebo (Mean±SE)		Mean Difference vs placebo (95%CI)	*P*-value
		No.	Mean±SE	No.	Mean±SE		
Congestion							
	Week 4	76	-0.08±0.99	37	-0.08±0.92	-0.09 (-0.41- 0.23)	0.5693
	Week 12	73	0.01±1.11	34	-0.24±1.02	0.17 (-0.15- 0.49)	0.2901
	Week 20	72	0.08±0.92	34	-0.24±0.99	0.26 (-0.02- 0.55)	0.0695
	Week 24	72	0.10±0.98	33	-0.12±0.86	0.20 (-0.12- 0.51)	0.2160
Daily cough							
	Week 4	76	-0.07±0.68	37	-0.05±0.78	-0.01 (-0.29- 0.26)	0.9192
	Week 12	73	0.14±0.63	34	0.00±0.60	0.16 (-0.08- 0.39)	0.1860
	Week 20	72	0.15±0.74	34	0.00±0.82	0.23 (-0.02- 0.48)	0.0676
	Week 24	72	0.18±0.66	33	-0.03±0.68	0.29 (0.05- 0.53)	0.0199
Sputum production							
	Week 4	76	-0.05±0.92	37	0.03±0.93	-0.10 (-0.43- 0.23)	0.5454
	Week 12	73	0.14±0.99	34	-0.03±0.83	0.17 (-0.17- 0.52)	0.3268
	Week 20	72	0.15±0.88	34	0.06±0.85	0.09 (-0.22- 0.40)	0.5655
	Week 24	72	0.22±0.92	33	0.03±0.64	0.18 (-0.13- 0.50)	0.2521
Sputum purulence							
	Week 4	76	-0.05±0.68	37	-0.17±0.70	0.06 (-0.19- 0.32)	0.6106
	Week 12	73	0.01±0.64	34	-0.21±0.74	0.15 (-0.11- 0.42)	0.2575
	Week 20	72	0.06±0.75	34	-0.21±0.74	0.20 (-0.09- 0.50)	0.1758
	Week 24	72	-0.09±0.81	33	-0.22±0.79	0.04 (-0.26- 0.35)	0.7818
Breathlessness on daily activity							
	Week 4	76	-0.03±0.67	37	-0.16±0.55	0.11 (-0.12- 0.35)	0.3351
	Week 12	73	0.03±0.85	34	-0.26±0.71	0.26 (-0.03- 0.56)	0.0805
	Week 20	72	0.11±0.83	34	-0.15±0.66	0.21 (-0.06- 0.49)	0.1275
	Week 24	72	0.08±0.87	33	-0.21±0.55	0.23 (-0.06- 0.53)	0.1239
Wheeze							
	Week 4	76	0.00±0.46	37	0.00±0.67	0.08 (-0.11- 0.26)	0.4187
	Week 12	73	-0.10±0.58	34	0.00±0.85	0.01 (-0.23- 0.25)	0.9323
	Week 20	72	0.07±0.48	34	-0.06±0.69	0.20 (0.00- 0.39)	0.0471

	Week 24	72	0.01±0.49	33	-0.03±0.64	0.10 (-0.09- 0.28)	0.3031
Chest pain							
	Week 4	76	-0.01±0.58	37	-0.03±0.50	0.00 (-0.20- 0.21)	0.9673
	Week 12	73	0.07±0.56	34	0.06±0.60	-0.01 (-0.21- 0.20)	0.9543
	Week 20	72	0.11±0.46	34	0.03±0.58	0.05 (-0.11- 0.22)	0.5324
	Week 24	72	0.06±0.53	33	-0.09±0.58	0.12 (-0.06- 0.31)	0.1909
Breathlessness on talking							
	Week 4	76	0.03±0.33	37	0.08±0.55	0.02 (-0.13- 0.18)	0.7797
	Week 12	73	0.01±0.46	34	0.15±0.70	-0.02 (-0.22- 0.19)	0.8720
	Week 20	72	-0.06±0.50	34	0.06±0.65	0.02 (-0.19- 0.23)	0.8430
	Week 24	72	-0.06±0.65	33	0.03±0.53	0.06 (-0.17- 0.29)	0.5969
Nocturnal cough							
	Week 4	76	0.03±0.59	37	-0.14±0.54	0.19 (-0.01- 0.39)	0.0568
	Week 12	73	0.10±0.50	34	0.03±0.58	0.09 (-0.09- 0.28)	0.3096
	Week 20	72	0.03±0.58	34	-0.03±0.72	0.09 (-0.15- 0.32)	0.4653
	Week 24	72	-0.01±0.62	33	0.00±0.71	0.01 (-0.23- 0.26)	0.9072

Mean changes from baseline in individual QoL-B-RSS symptom domains between florensocatib and placebo in patient with low symptom burden (QoL-B-RSS≥60).

Abbreviations: QoL-B-RSS=Quality-of-Life Bronchiectasis Respiratory Symptoms Scale; SE: standard error; CI: confidence interval.

Table S6. Mean changes from baseline in individual QoL-B-RSS item scores at day 29 and day 85 in patients receiving inhaled tobramycin versus placebo in the TORNASOL trial. Related to Figure 5.

Items	High-symptom subgroup				Low-symptom subgroup			
	**Tobramycin (Mean±SE) n=77**	**Placebo (Mean±SE) n=63**	**Mean Difference vs placebo (95%CI)**	***P*-value**	**Tobramycin (Mean±SE) n=89**	**Placebo (Mean±SE) n=108**	**Mean Difference vs placebo (95%CI)**	***P*-value**
Day 29								
Congestion	0.72±0.08	0.15±0.09	0.58 (0.26-0.88)	<0.0001	0.26±0.06	0.05±0.06	0.21 (-0.02-0.43)	0.0894
Daily cough	0.59±0.08	0.11±0.09	0.48 (0.10-0.80)	0.0006	0.10±0.05	-0.12±0.05	0.22 (0.03-0.41)	0.0116
Sputum production	0.55±0.08	0.16±0.09	0.39 (0.08-0.70)	0.0056	0.23±0.06	-0.10±0.05	0.32 (0.11-0.54)	0.0005
Sputum purulence	0.47±0.07	0.09±0.07	0.38 (0.11-0.64)	0.0017	0.38±0.05	-0.11±0.05	0.48 (0.29-0.68)	<0.0001
Breathlessness on daily activity	0.35±0.08	0.10±0.09	0.25 (-0.08-0.51)	0.2755	0.13±0.06	-0.07±0.06	0.19 (-0.03-0.42)	0.1373
Wheeze	0.13±0.06	0.02±0.07	0.11 (-0.14-0.36)	>0.9999	-0.03±0.04	-0.00±0.04	-0.03(-0.18-0.12)	>0.9999
Chest pain	0.20±0.06	0.06±0.06	0.13 (-0.09-0.36)	0.7015	0.04±0.03	-0.03±0.03	0.07 (-0.05-0.20)	0.6790
Breathlessness on talking	0.33±0.08	0.05±0.09	0.28 (-0.03-0.59)	0.1108	-0.08±0.06	-0.03±0.05	-0.05(-0.25-0.15)	>0.9999
Nocturnal cough	0.20±0.07	0.03±0.08	0.17 (-0.12-0.45)	0.6834	0.03±0.05	-0.05±0.04	0.08 (-0.09-0.25)	>0.9999
Day 85								
Congestion	0.65±0.08	0.19±0.09	0.47 (0.15-0.79)	0.0010	0.729±0.06	0.05±0.06	0.25 (0.02-0.48)	0.0283
Daily cough	0.54±0.08	0.23±0.09	0.32 (-0.02-0.65)	0.0737	0.14±0.05	-0.07±0.05	0.21 (0.02-0.40)	0.0217
Sputum production	0.58±0.08	0.26±0.09	0.32 (-0.01-0.64)	0.0666	0.26±0.06	-0.03±0.06	0.29 (0.07-0.51)	0.0038
Sputum purulence	0.43±0.07	0.09±0.08	0.34 (0.05-0.62)	0.0106	0.44±0.06	-0.02±0.05	0.46 (0.26-0.66)	<0.0001
Breathlessness on daily activity	0.49±0.09	0.24±0.10	0.25 (-0.10-0.60)	0.3520	0.13±0.06	-0.01±0.06	0.14 (-0.08-0.37)	0.5471
Wheeze	0.11±0.07	0.15±0.07	-0.04 (-0.30-0.22)	>0.9999	-0.04±0.04	0.03±0.04	-0.06 (-0.21-0.09)	>0.9999
Chest pain	0.23±0.06	-0.02±0.07	0.25 (0.01-0.49)	0.0401	0.11±0.04	-0.00±0.03	0.12 (-0.01-0.24)	0.0984
Breathlessness on talking	0.34±0.08	0.06±0.09	0.27 (-0.05-0.59)	0.1478	-0.03±0.06	-0.08±0.05	0.05 (-0.15-0.23)	>0.9999
Nocturnal cough	0.22±0.08	0.28±0.08	-0.06 (-0.35-0.24)	>0.9999	0.09±0.05	0.02±0.04	0.06 (-0.11-0.24)	>0.9999

The mean difference represents the treatment effect between tobramycin and placebo. And the estimates have been adjusted for baseline symptom score. High-symptom: QoL-B-RSS<60; low-symptom: QoL-B-RSS≥60. QoL-B-RSS: Quality-of-Life Bronchiectasis Respiratory Symptoms Scale; SE: standard error; CI: confidence interval.

Table S7. Individual items comprising the Respiratory Symptom Domain of the Quality-of-Life Bronchiectasis (QoL-B) (questions 29 - 37). Related to STAR Methods.

Q29 Have you felt congestion (fullness) in your chest?	1.A lot	2.A moderate moment	3.A little	4.Not at all
Q30 Have you been coughing during the day?	1.A lot	2.A moderate moment	3.A little	4.Not at all
Q31 Have you had to cough up sputum?	1.A lot	2.A moderate moment	3.A little	4.Not at all
Q32 Has your sputum been mostly:	1.Green with traces of blood or brownish-dark	2.Yellowish-green	3.Clear to yellow	4.Clear
Q33 Have you had shortness of breath when being active, such as when doing housework or gardening?	1.Always	2.Often	3.Sometimes	4.Never
Q34 Have you had wheezing?	1.Always	2.Often	3.Sometimes	4.Never
Q35 Have you had chest pain?	1.Always	2.Often	3.Sometimes	4.Never
Q36 Have you had shortness of breath when talking?	1.Always	2.Often	3.Sometimes	4.Never
Q37 Have you woken during the night because you were coughing?	1.Always	2.Often	3.Sometimes	4.Never

Supplementary figures

Figure S1.

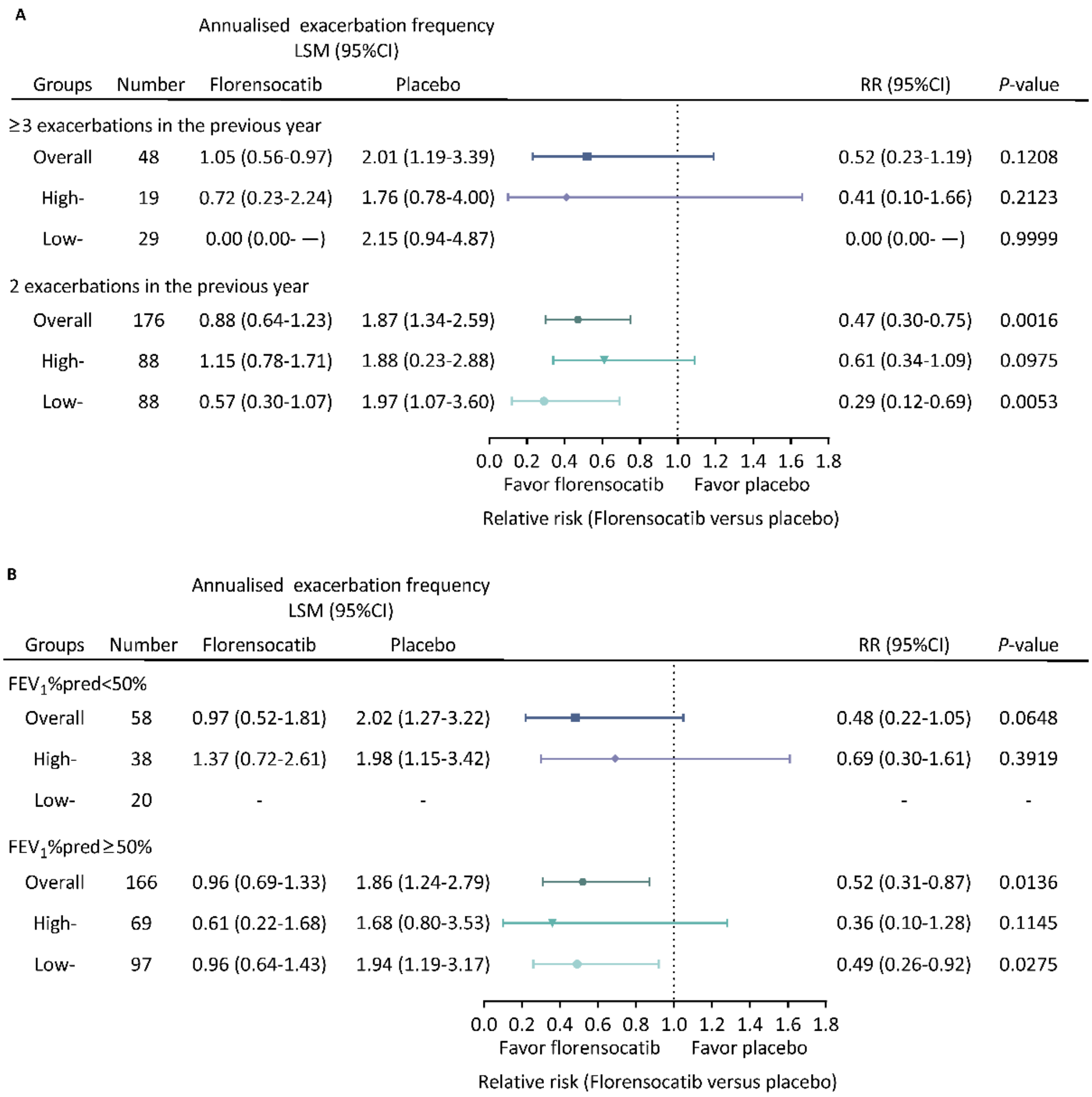

Figure S1. Efficacy of florensocatib in reducing annualised exacerbation rate stratified by symptom burden across disease severity strata in SAVA-BE trial. Related to Figure 1 and Figure 2.

The forest plots show the efficacy of florensocatib versus placebo in reducing annualised frequency of bronchiectasis exacerbation throughout the treatment period of all patients and those with high- or low-symptom burden stratified by different disease severity assessed by exacerbations in the previous one year (≥3 or 2) (A) and FEV1% of predicted (<50% or ≥50%) (B) in the SAVE-BE trial. The annualised exacerbation frequency per person-year is shown as least-squares mean (LSM) with 95% confidence interval (CI). Squares, dots or triangles represent the adjusted mean differences and horizontal lines represent the 95% confidence interval (CI). The relative risk (RR) for the annualised exacerbation frequency is reported for florensocatib versus placebo. Estimates <1 favor florensocatib; >1 favor placebo. Estimates are adjusted for the exacerbation frequency within the previous year (cut-off: 3 events).

High-symptom burden group: QoL-B-RSS <60; low-symptom burden group: QoL-B-RSS ≥60.

Abbreviations: QoL-B-RSS: Quality-of-Life Bronchiectasis Respiratory Symptoms Scale; RR: relative

risk; CI: confidence interval.

Figure S2.

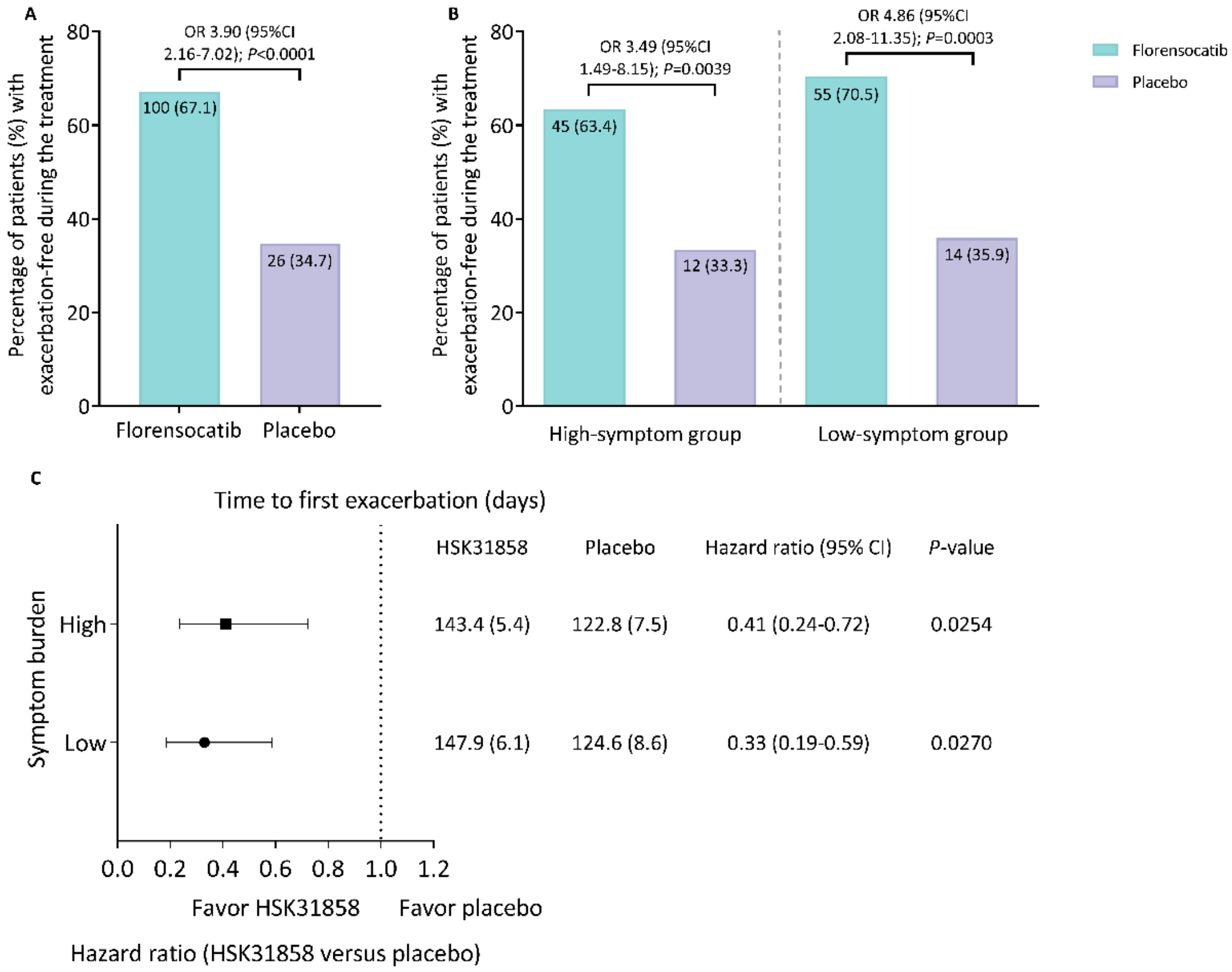

Figure S2. Efficacy of florensocatib in remaining bronchiectasis exacerbation-free and prolonging the time to the first exacerbation, stratified by symptom burden, in the SAVE-BE trial. Related to Figure 1 and Figure 2.

(A) The percentage of patients who remained exacerbation-free during the treatment period according to the SAVE-BE trial group allocation. (B) The percentage of patients who remained exacerbation-free during the treatment period for patients with high-symptom burden and those with low-symptom burden, respectively. The green bars refer to florensocatib, and the purple bars represent placebo. The odd ratio (OR) for remaining exacerbation-free are reported here for florensocatib versus placebo.

(C) The time to the first exacerbation during the treatment period for patients with high- or low-symptom burden, respectively. Time to the first exacerbation (days [standard error]) for each treatment groups in different symptom subgroups over 24 weeks are showed. Squares and dots represent hazard ratio (HR) and horizontal lines represent 95% confidence interval (CI). The HR with 95%CI for the time to the first exacerbation are also reported here for florensocatib versus placebo.

High-symptom burden group: QoL-B-RSS<60; low-symptom burden group: QoL-B-RSS≥60.

Abbreviations: QoL-B-RSS: Quality-of-Life Bronchiectasis Respiratory Symptoms Scale; OR: odd ratio; CI: confidence interval.

Figure S3.

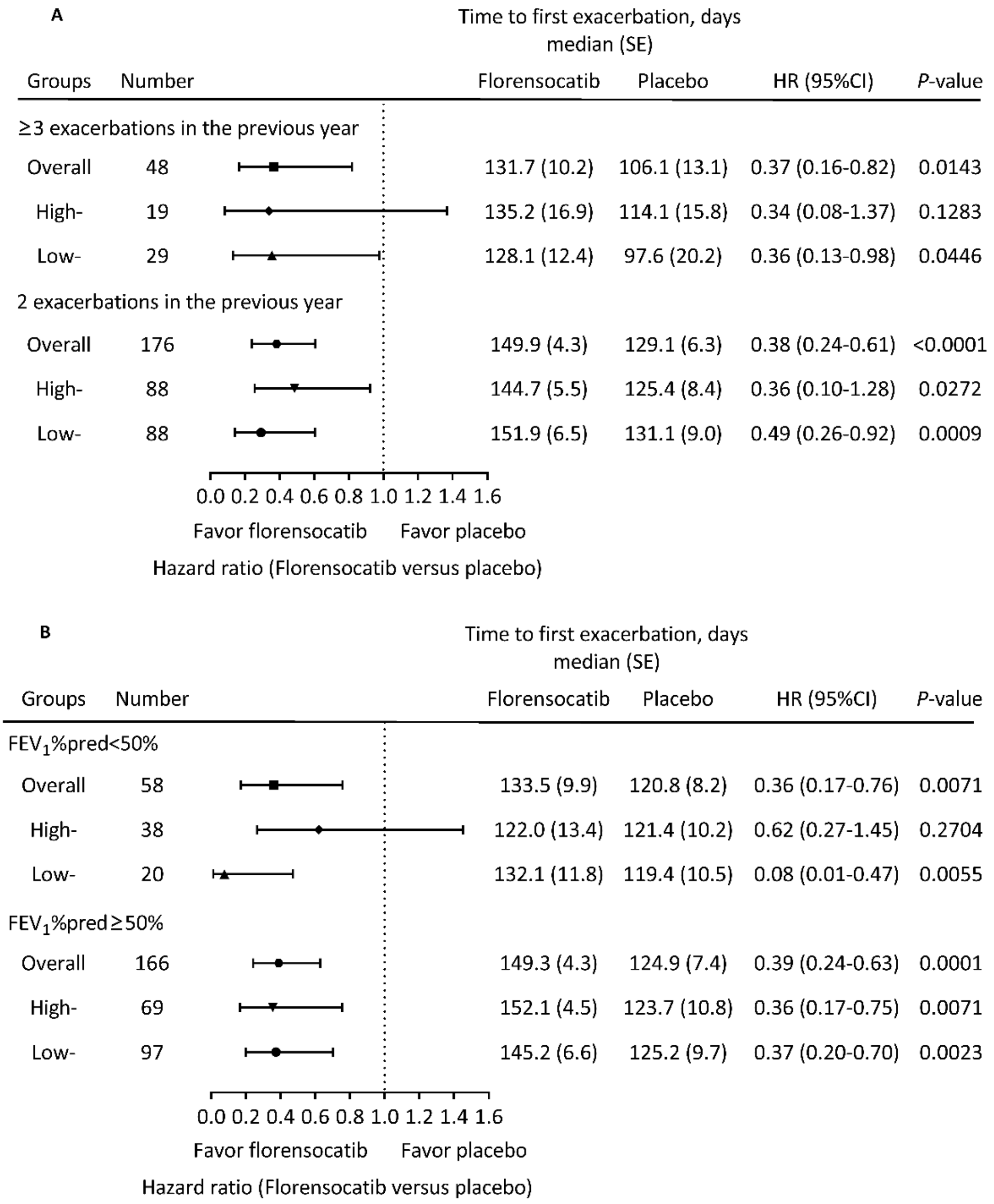

Figure S3. Efficacy of florensocatib in prolonging the time to the first exacerbation stratified by symptom burden across disease severity strata in SAVA-BE trial. Related to Figure 1 and Figure 2.

The time to the first exacerbation during the treatment period for all patients and those with high- or low-symptom burden stratified by different disease severity assessed by exacerbations in the previous year (≥3 or 2) (A) and FEV1% predicted (<50% or ≥50%) (B) in the SAVE-BE trial. Time to the first exacerbation (median days [standard error]) for each treatment groups in different subgroups over 24 weeks are showed. Squares, dots or triangles represent hazard ratio (HR) and horizontal lines represent 95% confidence interval (CI). The HR with 95%CI for the time to the first exacerbation are also reported here for florensocatib versus placebo.

High-symptom burden group: QoL-B-RSS<60; low-symptom burden group: QoL-B-RSS≥60.

Abbreviations: QoL-B-RSS: Quality-of-Life Bronchiectasis Respiratory Symptoms Scale; HR: hazard ratio; CI: confidence interval.

Figure S4.

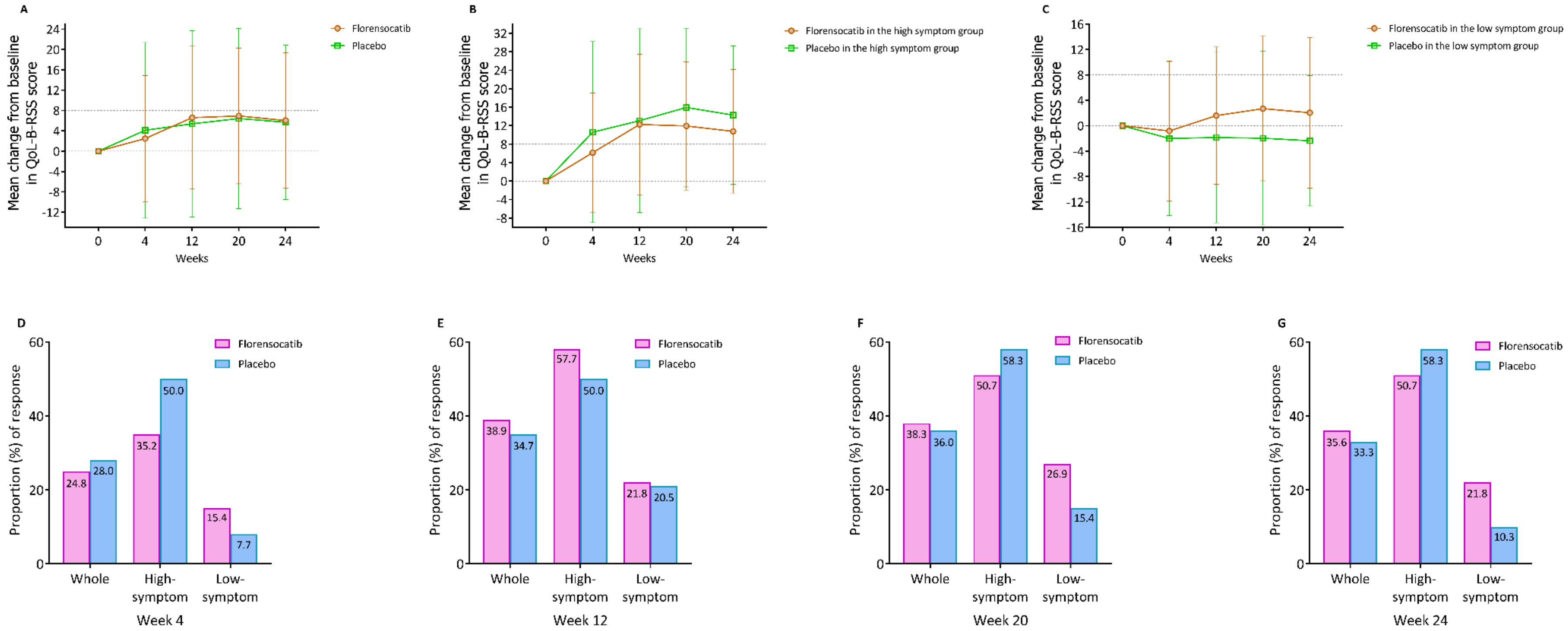

Figure S4. Change in QoL-B-RSS from baseline and the proportion of patients achieving a clinically meaningful improvement in QoL-B-RSS in the SAVE-BE trial. Related to Figure 1 and Figure 2.

(A-C) Mean change from baseline for QoL-B-RSS in whole patients and patients stratified by baseline symptom burden assessed by QoL-B-RSS and treatment arm. Panel A represents the whole patients in the SAVE-BE trial. Panel B stands for those with high symptom burden and Panel C indicates those with low symptom burden. Time point: Week 0 (baseline), 4, 12, 20 and 24.

(D-G) Bar charts display the percentage of patients with improvement (increase) from baseline in QoL-B-RSS score of ≥8 points (established MCID threshold for the

respiratory symptoms domain) at week 4, 12, 20 and 24, respectively. All $P>0.05$ for comparison between florensocatib and placebo.

High symptom burden: <60; low symptom burden: ≥60. Abbreviations: QoL-B-RSS=Quality-of-Life Bronchiectasis Respiratory Symptom Scale. MCID: minimun clinically important difference.

Figure S5.

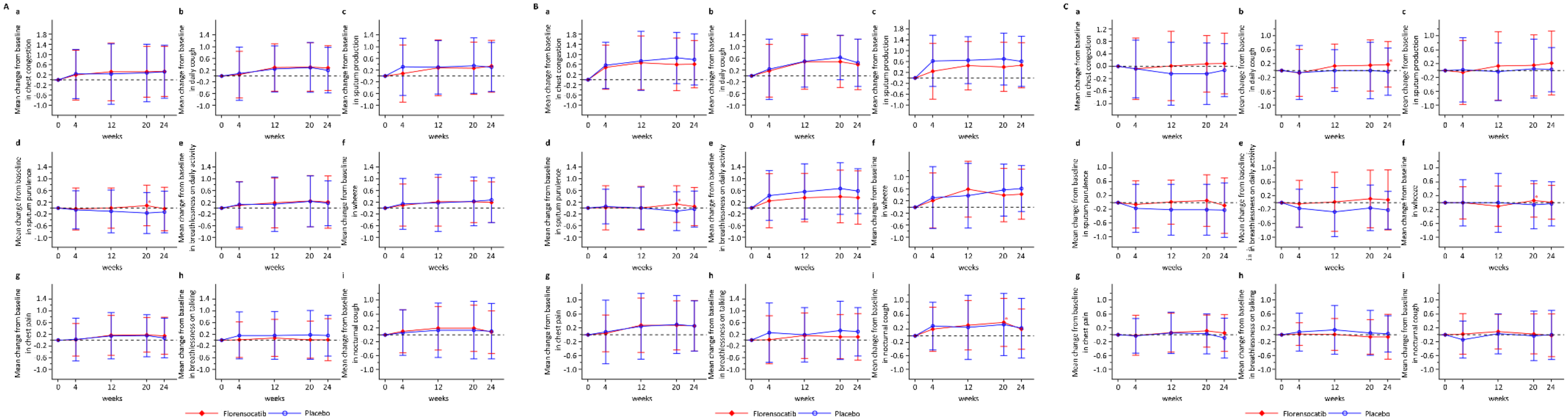

Figure S5. Changes from baseline in individual respiratory symptoms of QoL-B-RSS in patients receiving florensocatib versus placebo in the SAVE-BE trial. Related to Figure 1 and Figure 2.

(A) Mean changes from baseline in individual QoL-B-RSS symptom domains are presented for the florensocatib group (red line) and the placebo group (blue line) in patients receiving florensocatib versus placebo.

(B) Mean changes from baseline in individual QoL-B-RSS symptom domains are presented for the florensocatib group (red line) and the placebo group (blue line) in patients with high-symptom burden receiving florensocatib versus placebo.

(C) Mean changes from baseline in individual QoL-B-RSS symptom domains are presented for the florensocatib group (red line) and the placebo group (blue line) in patients with low-symptom burden receiving florensocatib versus placebo.

Panels show the following domains: a) chest congestion; b) daily cough; c) sputum production; d) sputum purulence; e) breathlessness on daily activity; f) wheeze; g) chest pain; h) breathlessness on talking; and i) nocturnal cough. Abbreviations: QoL-B-RSS=Quality-of-Life Bronchiectasis Respiratory Symptoms Scale.

Figure S6.

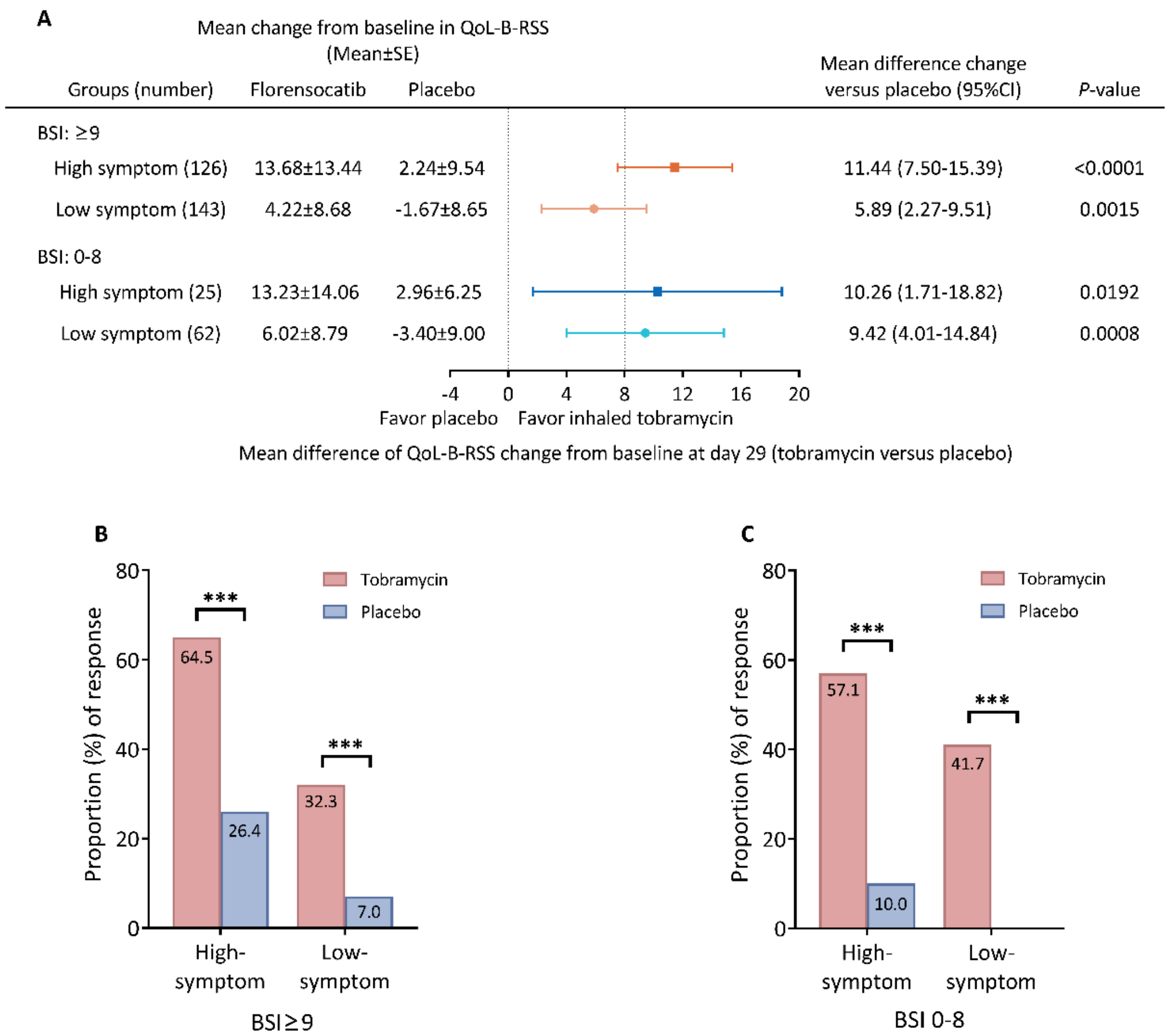

Figure S6. Change in QoL-B-RSS from baseline and the proportion of patients achieving a clinically meaningful improvement in QoL-B-RSS in patients with different symptom burden stratified by disease severity in TORNASOL trial. Related to Figure 3 and Figure 4.
(A) Mean change from baseline for QoL-B-RSS of all patients and those with high- or low-symptom burden stratified by different disease severity assessed by bronchiectasis severity index (BSI) and treatment arm at day 29 (the primary outcome time point). (B-C) Bart charts display the percentage of patients with an improvement (increase) from baseline in QoL-B-RSS score of ≥8 points for those with BSI ≥9 and BSI 0-8, respectively, at day 29. ***: P<0.0001.
Severe disease: BSI≥9; Moderate and mild disease: BSI 0-8; High-symptom burden: <60; low-symptom burden: ≥60. Abbreviations: QoL-B-RSS=Quality-of-Life Bronchiectasis Respiratory Symptoms Scale. MCID: minimum clinically important difference.

Figure S7.

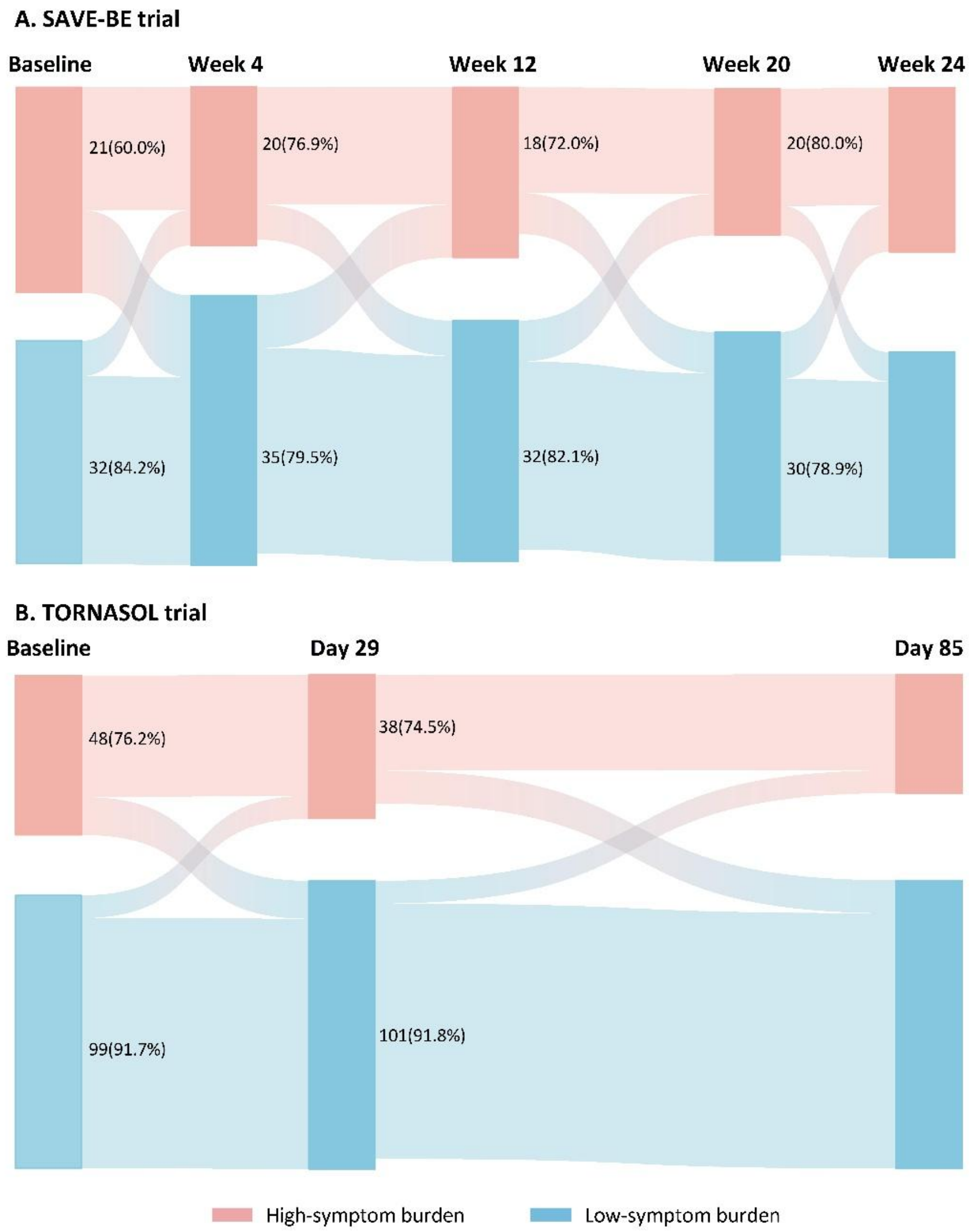

Figure S7. Change of symptom burden subgroups in placebo arm in SAVE-BE trial and TORNASOL trial. Related to Figure 1 and Figure 2. Related to Figure 2 and Figure 4.

Sankey diagram illustrating the transitions in symptom burden subgroups during the trial period for patients with bronchiectasis in placebo arm in (A) SAVE-BE trial and (B) TORNASOL trials. Vertical nodes represent the distribution of patients across two symptom burden subgroups at each study visit: high- (QoL-B-RSS<60, red) and low- (QoL-B-RSS≥60, blue) symptom burden. The width of connecting flows is proportional to the number of patients transitioning between subgroups or remaining in the same subgroup from one time point to the next. Numerical labels on the flows indicate the absolute number and percentages of patients for each persistence.
