# Supplementary material for: Symptoms and treatment response to florensocatib and inhaled tobramycin in bronchiectasis: Post hoc analysis of two randomized trials
Source: Cell Rep Med. 2026 Aug 13;7(9):102995. doi: 10.1016/j.xcrm.2026.102995 (PMC13589483; doi:10.1016/j.xcrm.2026.102995)
Supplement: Supplementary file 2 [file mmc2.pdf]

Cell Reports
Medicine

Article

Symptoms and treatment response to florensocatib and inhaled tobramycin in bronchiectasis: Post hoc analysis of two randomized trials

Graphical abstract

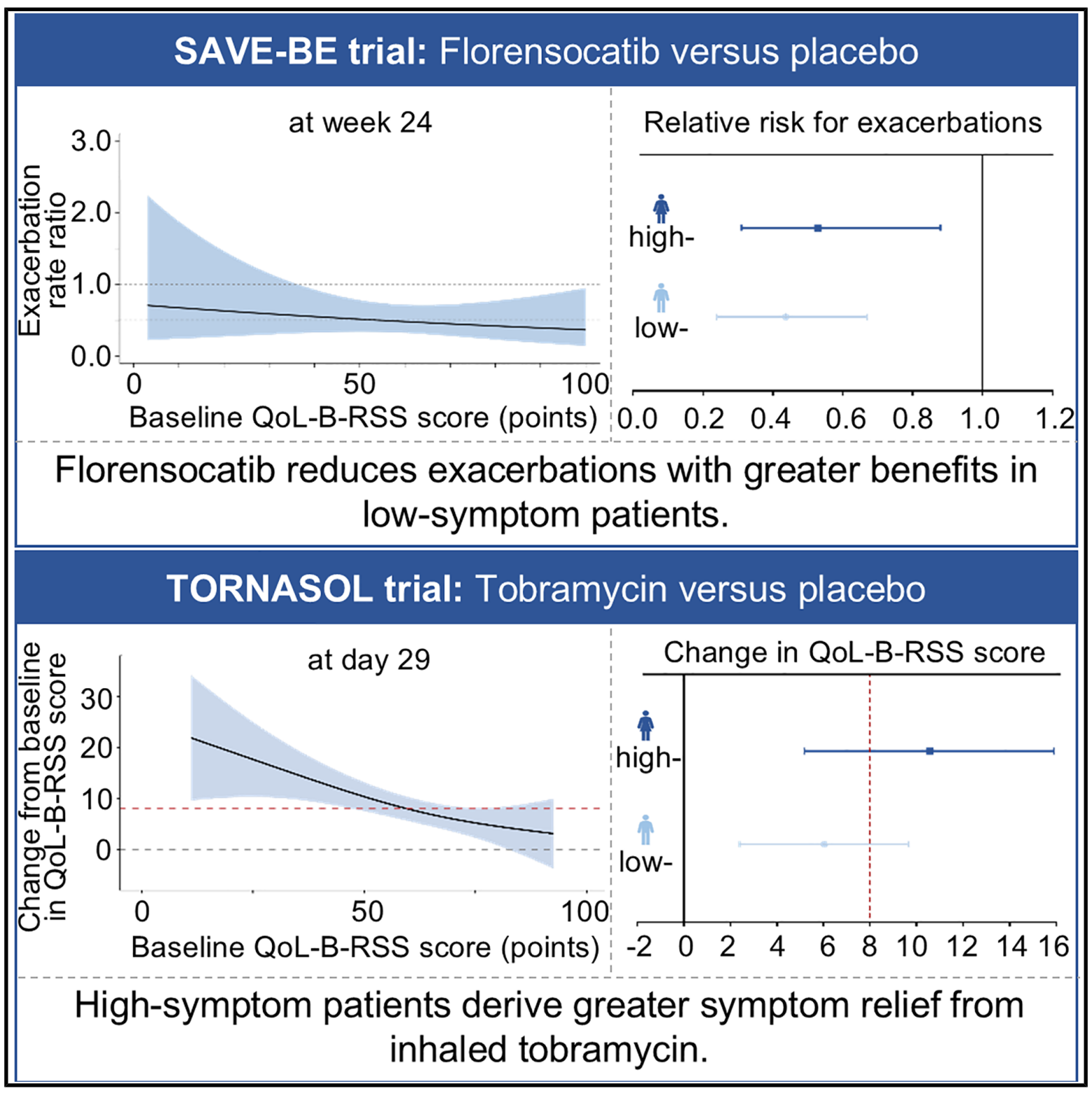

Authors

Yong-Hua Gao (高永华), Xue-Shan Li (李雪杉), Xiao-Li Sun (孙晓丽), Fang-Qiong Li (李芳琼), Lu-Yin Sun (孙露银), Han-Mo Liu (刘翰墨), Wei-Jie Guan (关伟杰)

Correspondence

gaoyonghuahust@163.com (Y.-H.G.),
battery203@163.com (W.-J.G.)

In brief

Gao et al. show that baseline symptom burden predicts differential treatment responses in bronchiectasis. Florensocatib reduces exacerbations more in low-symptom patients, while inhaled tobramycin improves symptoms more in high-symptom patients. Symptom burden may serve as a simple tool for personalized therapy.

Highlights

- Baseline symptom burden predicts differential treatment responses in bronchiectasis
- Florensocatib reduces exacerbations, with greater benefits in low-symptom patients
- High-symptom patients derive greater symptom relief from inhaled tobramycin
- Symptom burden serves as a simple, practical tool to guide personalized therapy

Gao et al., 2026, Cell Reports Medicine 7, 102995
September 15, 2026 © 2026 The Author(s). Published by Elsevier Inc.
https://doi.org/10.1016/j.xcrm.2026.102995

CellPress

Cell Reports Medicine

CellPress
OPEN ACCESS

Article

Symptoms and treatment response to florensocatib and inhaled tobramycin in bronchiectasis: Post hoc analysis of two randomized trials

Yong-Hua Gao (高永华),[1,6,*] Xue-Shan Li (李雪杉),[1,6] Xiao-Li Sun (孙晓丽),[1] Fang-Qiong Li (李芳琼),[2] Lu-Yin Sun (孙露银),[2] Han-Mo Liu (刘翰墨),[2] and Wei-Jie Guan (关伟杰)[3,4,5,7,*]

[1]Department of Respiratory and Critical Care Medicine, Shanghai Pulmonary Hospital, Tongji University School of Medicine, Shanghai, P.R. China

[2]Department of Clinical Medicine, Haisco, Chengdu, China

[3]State Key Laboratory of Respiratory Disease, National Clinical Research Center for Respiratory Disease, National Center for Respiratory Medicine, Joint International Research Laboratory of Respiratory Health, Guangdong Basic Research Center of Excellence for Respiratory Medicine, Department of Allergy and Clinical Immunology, Department of Respiratory and Critical Care Medicine, Guangzhou Institute of Respiratory Health, the First Affiliated Hospital of Guangzhou Medical University, Guangzhou 510163, P.R. China

[4]China-Portugal Artificial Intelligence and Public Health Technologies Joint Laboratory, Guangdong-Hong Kong-Macao Joint Laboratory of Respiratory Infectious Diseases, Guangdong Provincial Key Laboratory of Respiratory Disease Research, Guangzhou Medical University, Guangzhou 510163, P.R. China

[5]Shanxi Bethune Hospital, Taiyuan 050081, P.R. China

[6]These authors contributed equally

[7]Lead contact

*Correspondence: gaoyonghuahust@163.com (Y.-H.G.), battery203@163.com (W.-J.G.)

https://doi.org/10.1016/j.xcrm.2026.102995

SUMMARY

Inhaled antibiotics and DPP-1 inhibitors improve clinical outcomes in bronchiectasis, but whether baseline symptom burden predicts differential treatment responses remains unclear. In this post hoc analysis of two multicenter randomized trials (SAVE-BE, $n = 224$; TORNASOL, $n = 357$), we evaluate the association between baseline Quality of Life-Bronchiectasis Respiratory Symptom Scale (QoL-B-RSS) and treatment effects of florensocatib and inhaled tobramycin. In SAVE-BE, florensocatib reduces exacerbation rates versus placebo (relative risk [RR], 0.47; 95% confidence interval [CI], 0.33–0.67; $p < 0.0001$), with RRs of 0.53 and 0.40 observed in patients with high and low symptom burdens, respectively, but no significant symptomatic improvement. In TORNASOL, tobramycin produces clinically meaningful QoL-B-RSS improvements (exceeding the 8-point cutoff in high-symptom patients) and ameliorates bronchitic symptoms, with greater benefits in those with higher baseline symptom burden. These hypothesis-generating findings suggest that baseline symptom burden may identify differential responses to anti-inflammatory versus anti-infective therapies in bronchiectasis and support its potential as a simple, practical stratification tool to guide personalized treatment.

INTRODUCTION

Bronchiectasis in adults is a chronic airway disease characterized by incompletely reversible bronchial dilatation, impaired mucociliary clearance, recurrent infections, and predominantly neutrophilic inflammation.[1,2] Patients experience significant symptom burden, particularly chronic cough, sputum production, and dyspnea, along with frequent exacerbations that impair the health-related quality of life, accelerate lung function decline, and increase mortality risk.[1,3,4] International guidelines prioritize symptom improvement and exacerbation reduction as the primary therapeutic goals after addressing the underlying etiologies.[5,6]

Management of bronchiectasis includes airway clearance techniques, mucoactive drugs, inhaled antibiotics, and macrolides, all of which aim to alleviate symptoms and prevent from exacerbations.[6–8] Inhaled antibiotics, such as tobramycin, reduced exacerbation risk by 20%–40% and improved bronchitic symptoms (e.g., cough, sputum production, and chest congestion) in randomized controlled trials, particularly in patients with high bacterial burden.[8–12] However, quantitative bacterial load has not been routinely assessed in clinical practice, limiting its utility as the first-line biomarker for treatment decision-making. The Quality of Life-Bronchiectasis Respiratory Symptom Scale (QoL-B-RSS) is a validated tool that quantifies daily symptoms, independently predicting future exacerbation risks in large cohorts (e.g., EMBARC registry, >9,000 patients) after adjusting for prior exacerbations and disease severity.[4,13] High symptom burden predicts more favorable responses to inhaled mannitol and macrolides,[13,14] but its role in guiding inhaled antibiotic therapy is unexplored.

© 2026 The Author(s). Published by Elsevier Inc.
This is an open access article under the CC BY license (http://creativecommons.org/licenses/by/4.0/).

Table 1. Baseline characteristics of adults with bronchiectasis and stratified analysis according to baseline QoL-B-RSS score in the SAVE-BE trial

Characteristics	Total (*N* = 224)	High symptom burden (*n* = 107)	Low symptom burden (*n* = 117)	*p* value
Age, y	56.1 ± 12.3	58.0 ± 13.2	54.3 ± 11.2	0.0014
Gender, *n* (%)	–	–	–	0.1678
Male	88 (39.3%)	37 (34.6%)	51 (43.6%)	–
Female	136 (60.7%)	70 (65.4%)	66 (56.4%)	–
Body mass index (kg/m^2)	22.4 ± 3.3	22.4 ± 3.5	22.4 ± 3.2	0.7087
Smoking state, *n* (%)	–	–	–	0.2177
Never smoker	193 (86.2%)	96 (89.7%)	97 (82.9%)	–
Ex-smoker	25 (11.2%)	10 (9.3%)	15 (12.8%)	–
Smoker	6 (2.7%)	1 (0.9%)	5 (4.3%)	–
FEV_1% predicted	70.2 ± 24.7	62.2 ± 23.2	77.6 ± 2.4	<0.0001
Exacerbations in preceding year, median (IQR)	2 (0)	2 (0)	2 (0)	0.1916
≥ 3 exacerbations in the previous year, *n* (%)	48 (21.4%)	19 (17.8%)	29 (24.8%)	0.2003
QoL-B-RSS score	60.6 ± 17.5	46.0 ± 11.7	73.9 ± 9.3	<0.0001
24-h sputum weight (g), median (IQR)	15.3 (21.2)	19.0 (26.5)	13.2 (14.6)	0.0018
Sputum purulence score	4.8 ± 1.7	5.2 ± 1.6	4.5 ± 1.8	0.0013
History of asthma, *n* (%)	19 (8.5%)	12 (11.2%)	7 (6.0%)	0.1604
History of COPD, *n* (%)	59 (26.3%)	36 (33.6%)	23 (19.7%)	0.0176
Sputum neutrophil elastase concentration, *n* (%)	–	–	–	0.0065
Less than the lower limit of detection	7 (3.1%)	0 (0%)	7 (6.0%)	–
Lower limit of detection to ≤20 μg/mL	94 (42.0%)	41 (38.3%)	53 (45.3%)	–
>20 μg/mL	119 (53.1%)	65 (60.7%)	54 (46.2%)	–
Medications, *n* (%)	–	–	–	–
Bronchodilators	56 (25.0%)	43 (40.2%)	13 (11.1%)	<0.0001
Long-term macrolides	12 (5.4%)	7 (6.5%)	5 (4.3%)	0.4513
Inhaled corticosteroids	13 (5.8%)	7 (6.5%)	6 (5.1%)	0.6512

All values are mean ± SD or *n* (%) unless otherwise stated.
High symptom burden: QoL-B-RSS<60; low-symptom burden: QoL-B-RSS≥60.
QoL-B-RSS, Quality-of-Life Bronchiectasis Respiratory Symptom Scale; FEV_1, forced expiratory volume in one second; COPD, chronic obstructive pulmonary disease; IQR, interquartile range.

Anti-inflammatory therapies target neutrophilic inflammation where neutrophil serine proteases degrade airway structures and promote mucus hypersecretion.[15,16] Dipeptidyl peptidase-1 (DPP-1) inhibitors brensocatib and florensocatib (HSK31858) blocked NSP activation within bone marrow neutrophils, mitigating inflammation without immunosuppression.[17–20] The phase 3 ASPEN trial demonstrated significant exacerbation reductions (rate ratio: 0.79–0.81) and slower forced expiratory volume in one second (FEV_1) decline with brensocatib treatment, but symptom improvements were below the QoL-B-RSS minimal clinically important difference (MCID) of 8 points.[21,22] Similarly, in the phase 2 SAVE-BE trial, florensocatib reduced exacerbations without significant symptomatic benefits.[20] The composite nature of QoL-B-RSS, which includes both bronchitic and non-bronchitic symptoms (e.g., dyspnea and wheezing), may have limited its sensitivity to anti-inflammatory effects.

Whether the baseline symptom burden can help identify patients more likely to benefit from DPP-1 inhibitors or inhaled antibiotics remains unclear. This post hoc analysis of the SAVE-BE and TORNASOL trials evaluated whether the baseline QoL-B-RSS is associated with differential treatment responses to florensocatib and inhaled tobramycin in adults with bronchiectasis.

RESULTS

Outcomes of the SAVE-BE trial

Baseline characteristics have been reported previously. Briefly, 224 patients (mean age, 56.1 years; 60.7% female, mean FEV_1% predicted, 70.2%) were randomized. Of these, 117 (52.2%) had low symptom burden (QoL-B-RSS ≥60) and 107 (47.8%) had high symptom burden (<60) (Table S1). Compared with patients with low symptom burden, those with high symptom burden demonstrated more severe disease, including lower FEV_1% predicted, higher sputum neutrophil elastase levels, higher sputum purulence scores, higher 24-h sputum volume, a higher proportion with a history of chronic obstructive pulmonary disease, and more frequent use of bronchodilators (Table 1).

Generalized additive models with negative binomial distribution demonstrated the relationship between QoL-B-RSS and

CellPress
OPEN ACCESS

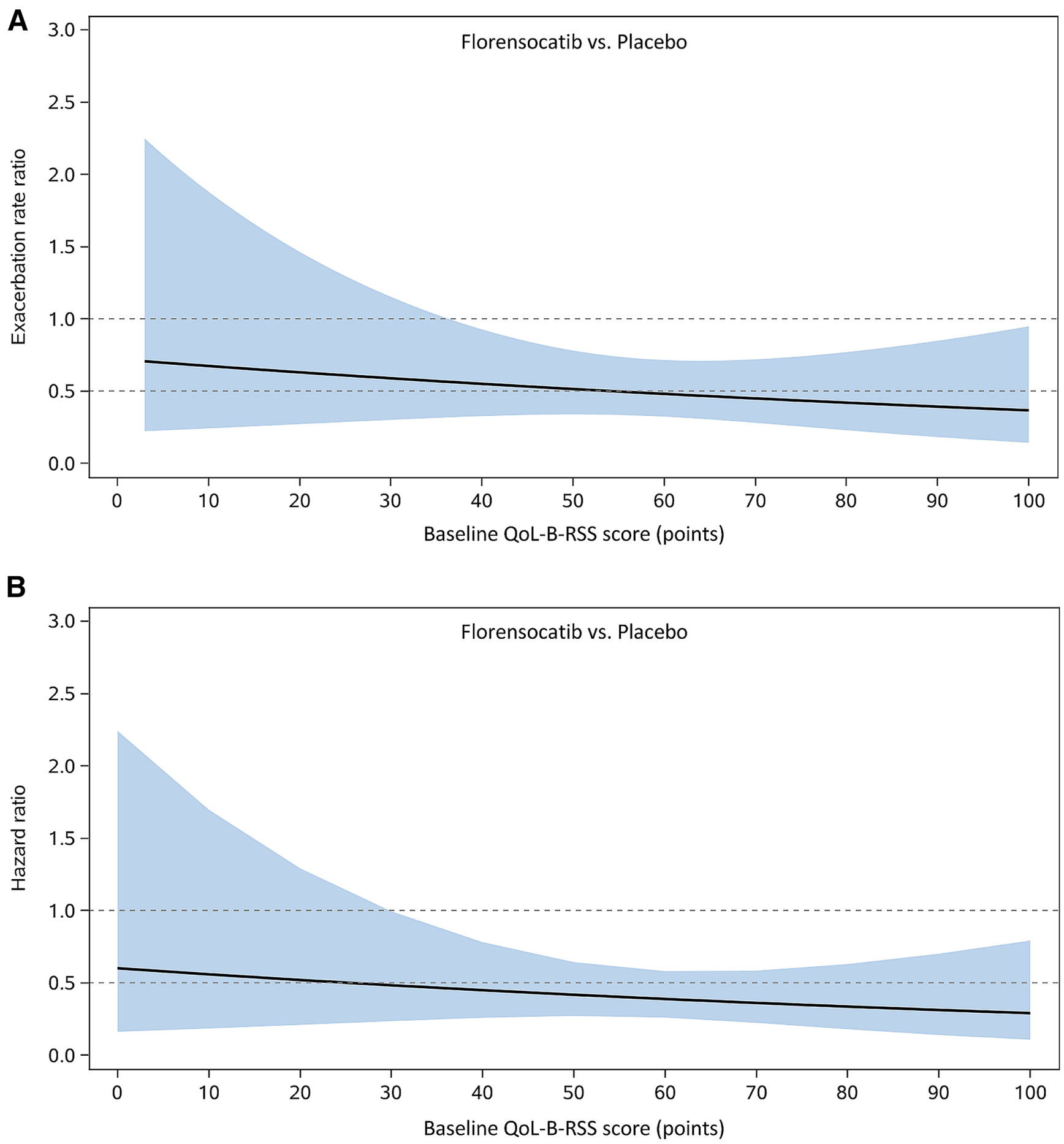

Figure 1. Relationship between baseline QoL-B-RSS score and exacerbation outcomes in the SAVE-BE trial

The estimated rate ratios between the treatment and placebo arms for different QoL-B-RSS, with 95% confidence intervals (CIs).

(A) Exacerbation rate reduction (rate ratio) for florensocatib versus control treatment effects, stratified by baseline QoL-B-RSS. The shaded area represents the 95% CIs. Dashed line equaled to 1.0 denotes no treatment effect (exacerbation rate ratio of 1.0); values < 1.0 represent an improvement of florensocatib versus control, whereas values > 1.0 represent a worsening of florensocatib versus control throughout the studies. A decrease of 0.5 in the exacerbation rate ratio (dashed line equaled to 0.5) represents a 50% reduction in the exacerbation rate.

(B) Time to the first exacerbation (hazard ratio) for florensocatib versus control treatment effects, stratified by the baseline QoL-B-RSS symptom score. Shaded area represents 95% CIs. Dashed line equaled to 1.0 denotes no treatment effect (hazard ratio of 1.0); values < 1.0 represent prolonged median time to the first exacerbation of florensocatib versus control, whereas values > 1.0 represent shortened median time to the first exacerbation of florensocatib versus control throughout the studies. A decrease of 0.5 in the hazard ratio (dashed line equaled to 0.5) represents a 50% prolongation of time to the first exacerbation.

the exacerbation frequency (Figure 1A). Florensocatib significantly reduced the exacerbation frequency versus placebo (relative risk [RR], 0.47; 95% confidence interval [CI], 0.33–0.67; $p < 0.0001$), with comparable reductions in low-symptom-burden (RR, 0.40; 95% CI, 0.24–0.67; $p = 0.0005$) and high-symptom-burden (RR, 0.53; 95% CI, 0.31–0.88; $p = 0.0139$) subgroups (Figure 2). The treatment effect of florensocatib on exacerbation frequency according to the baseline QoL-B-RSS was not modified by the disease severity, whether assessed by FEV_1% predicted or prior exacerbation history (*P* for interaction [$P_{interaction}$] = 0.72 and 0.48, respectively). Stratified analyses by prior exacerbation history or baseline FEV_1% predicted yielded similar estimates (Figure S1).

Among patients with low symptom burden, 55 (70.5%) in the florensocatib group versus 14 (35.9%) in the placebo group remained exacerbation free at 24 weeks (odds ratio [OR], 4.86;

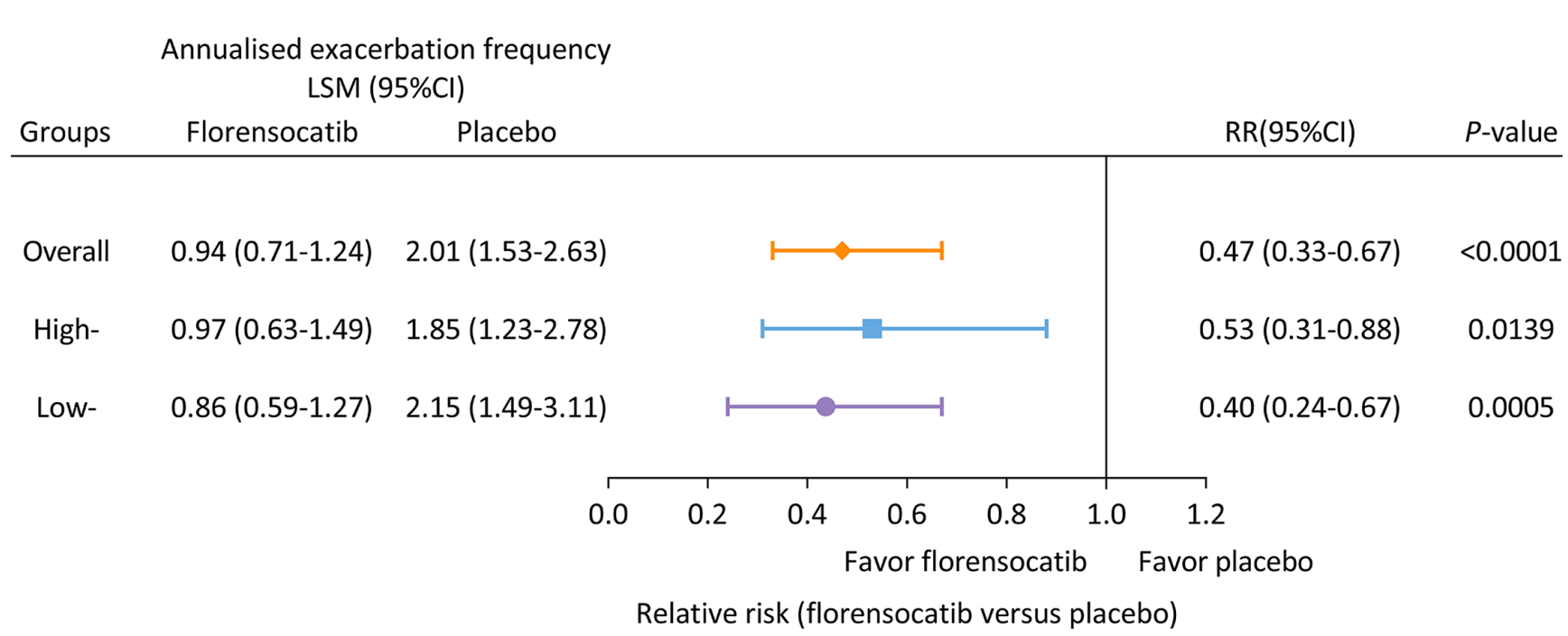

Figure 2. Efficacy of florensocatib in reducing the annualized exacerbation frequency risk stratified by symptom burden in the SAVE-BE trial

The forest plot shows the efficacy of florensocatib versus placebo in reducing the annualized frequency of bronchiectasis exacerbation throughout the treatment period of all patients and those with high or low symptom burden in the SAVE-BE trial. The annualized exacerbation frequency per person-year is shown as LSM with 95% CI. Squares, dots, or diamonds represent the adjusted mean differences, and horizontal lines represent the 95% CI. Orange, blue, and purple hcolors correspond to all patients, patients with igh symptom burden, and those with low symptom burden respectively. The RR for the annualized exacerbation frequency is reported for florensocatib versus placebo. Estimates <1 favor florensocatib, whereas estimates >1 favor placebo. Estimates are adjusted for the exacerbation frequency within the previous year (cutoff: 3 events). High-symptom-burden group: QoL-B-RSS <60; low-symptom-burden group: QoL-B-RSS ≥60. QoL-B-RSS: Quality-of-Life Bronchiectasis Respiratory Symptom Scale; RR, relative risk; CI, confidence interval; LSM, least-squares mean.

95% CI, 2.08–11.35; p = 0.0003). Among patients with high symptom burden, 45 (63.4%) in the florensocatib group versus 12 (33.3%) in the placebo group were exacerbation free (OR, 3.49; 95% CI, 1.49–8.15; p = 0.0039) (Figures S2A and S2B).

The relationship between QoL-B-RSS and time to the first exacerbation was modeled using a generalized additive model with Cox model analysis (Figure 1B). The time to the first exacerbation was significantly prolonged with florensocatib compared with placebo in both low- (median, 148 vs. 125 days; hazard ratio [HR], 0.33; 95% CI, 0.19–0.59; p = 0.0270) and high-symptom-burden subgroups (143 vs. 123 days; HR, 0.41; 95% CI, 0.24–0.72; p = 0.0254) (Figure S2C). The treatment effect of florensocatib on the time to the first exacerbation stratified by the baseline QoL-B-RSS was not modified by FEV_1% predicted or prior exacerbation history ($P_{interaction}$ = 0.88 and 0.86, respectively). Stratified analyses by prior exacerbation history or baseline FEV_1% predicted yielded similar effects (Figure S3).

Florensocatib significantly reduced 24-h sputum weight (low symptom: least-squares mean [LSM] difference, −5.77 g; 95% CI, −10.93 to −0.62; p = 0.0286; high symptom: LSM difference, −10.89 g; 95% CI, −21.46 to −0.31; p = 0.0437) and sputum purulence scores (low symptom: LSM difference, −0.90; 95% CI, −1.64 to −0.20; p = 0.0133; high symptom: −0.70; 95% CI, −1.38 to 0.04; p = 0.0653) at 24 weeks (Table S2). However, FEV_1% predicted remained unchanged in either group at week 24 (Table S2).

No significant QoL-B-RSS improvements occurred in low- (LSM difference, 4.53; 95% CI, −0.19 to 9.25; p = 0.0598) or high-symptom-burden subgroups (LSM difference, −2.75; 95% CI, −8.91 to 3.41; p = 0.3770) (Table S2). Changes of QoL-B-RSS over the study period are presented in Figures S4A–S4C, with no significant differences. No significant difference in attaining the MCID was observed between the two groups over time (all p > 0.05) (Figures S4D–S4G). Individual symptoms, including chest congestion, daily cough, sputum production, sputum purulence, breathlessness during daily activities or talking, wheezing, chest pain, or nocturnal cough, consistently showed no significant changes throughout the trial in both symptom burden subgroups (Figure S5; Tables S3–S5).

Outcomes of the TORNASOL trial

Of 357 patients (178 assigned to tobramycin and 179 to the placebo group), 152 (42.6%) had high symptom burden and 205 (57.4%) had low symptom burden (Table S1). Overall, 34.7% were male, the mean FEV_1% was 61.8% predicted, and the mean QoL-B-RSS was 61.6 (Table 2).

When baseline QoL-B-RSS was analyzed as a continuous variable, inhaled tobramycin showed a greater symptomatic improvement compared with placebo as the QoL-B-RSS scores decreased (i.e., increase in symptom burden) (Figure 3). At day 29, tobramycin improved QoL-B-RSS versus placebo in both high- (mean difference, 10.56; 95% CI, 5.19–15.92; p < 0.0001) and low-symptom-burden groups (mean difference, 6.03; 95% CI, 2.39–9.67; p = 0.0001), which was sustained to day 85 (high: 8.13; 95% CI, 2.56–13.70; p = 0.0009; low: 6.03; 95% CI, 2.35–9.72; p = 0.0001) (Figure 4A). No modifying effect of disease severity assessed by bronchiectasis severity index (BSI) on the association between baseline QoL-B-RSS score and symptomatic changes was observed ($P_{interaction}$ = 0.40). Analyses stratified by the BSI yielded similar effects (Figure S6**)**. Significantly more patients in the tobramycin group achieved MCID for QoL-B-RSS (high: 63.5% vs. 23.8%, p < 0.001; low: 34.8% vs. 4.6%, p < 0.001) at day 29, sustained to day 85 (Figures 4B and 4C).

In patients with high symptom burden, tobramycin significantly improved chest congestion (mean difference, 0.58; 95% CI, 0.26–0.88; p < 0.0001), daily cough (mean difference, 0.48; 95% CI, 0.10–0.80; p = 0.0006), sputum production (mean difference, 0.39; 95% CI, 0.08–0.70; p = 0.0056), and purulence (mean difference, 0.38; 95% CI, 0.11–0.64; p = 0.0017) at day 29 (Figure 5; Table S6). In addition, patients with low symptom burden showed significant improvements in daily cough (mean difference, 0.22; 95% CI, 0.03–0.41; p = 0.0116), sputum production (mean difference, 0.32; 95% CI, 0.11–0.54; p = 0.0005), and purulence (mean difference, 0.48; 95% CI, 0.29–0.68; p < 0.0001) (Figure 5; Table S6).

Stability of symptom burden in the SAVE-BE and TORNASOL trials

In the placebo arms of both trials, symptom burden subgroup classification was stable over the study period (Figure S7). Approximately 75% of patients with high symptom burden at baseline remained in the high-symptom subgroup throughout study visits in both the SAVE-BE and TORNASOL trials. In contrast, stability was nominally greater in the low-symptom subgroup, with approximately 80% of the patients retaining their baseline classification in the SAVE-BE trial and >90% in the TORNASOL trial.

DISCUSSION

This post hoc analysis of the SAVE-BE and TORNASOL trials examined whether the baseline symptom burden, assessed by QoL-B-RSS, is associated with differential treatment responses to the DPP-1 inhibitor florensocatib and inhaled tobramycin in adults with bronchiectasis. Florensocatib significantly reduced the exacerbation rates, with estimates numerically favoring patients with lower symptom burden, but did not improve the overall or individual respiratory symptoms. In contrast, tobramycin produced clinically meaningful and sustained improvements in QoL-B RSS scores—particularly in those with high baseline symptom burden (exceeding the MCID)—and mitigated bronchitic symptom (cough, sputum volume and purulence) irrespective of the baseline symptom burden.

The lack of symptomatic improvement with florensocatib, despite robust effects on exacerbation reduction, is noteworthy and consistent with the findings from the phase 3 ASPEN trial of brensocatib,[21] which reported significant exacerbation reductions and non-significant QoL-B-RSS improvements (below the MCID). This dissociation may reflect several factors. First, the QoL-B-RSS is a composite quality-of-life measure[22] which includes both bronchitic and non-bronchitic symptoms (e.g., dyspnea and wheezing) that may be less responsive to therapies targeting neutrophil serine proteases. Domain-specific analyses confirmed minimal changes across individual symptom items. Second, exacerbations and respiratory symptoms may reflect

Table 2. Baseline characteristics of adults with bronchiectasis and stratified analysis according to baseline QoL-B-RSS score in the TORNASOL trial

Characteristics	Total (*N* = 357)	High symptom burden (*n* = 152)	Low symptom burden (*n* = 205)	*p* value
Age, y	53.6 ± 12.2	54.8 ± 12.7	52.8 ± 11.7	0.1302
Gender, *n* (%)	–	–	–	0.0480
Male	124 (34.7%)	44 (28.9%)	80 (39.0%)	–
Female	233 (65.3%)	108 (71.1%)	125 (61.0%)	–
Body mass index (kg/m^2)	21.7 ± 3.3	21.6 ± 3.7	21.7 ± 3.0	0.7695
Smoking state, *n* (%)	–	–	–	0.5390
Never smoker	303 (84.9%)	128 (85.3%)	175 (87.1%)	–
Ex-smoker	41 (11.5%)	20 (13.3%)	21 (10.4%)	–
Smoker	7 (2.0%)	2 (1.3%)	5 (2.5%)	–
BSI	11.0 ± 3.4	12.1 ± 3.2	10.3 ± 3.3	<0.0001
FEV_1% predicted	61.8 ± 22.2	55.4 ± 21.3	66.5 ± 21.6	<0.0001
Exacerbations in preceding year, median (IQR)	1 (1)	1 (1)	1 (0)	0.0005
QoL-B-RSS score	61.6 ± 14.0	48.2 ± 9.1	71.5 ± 6.9	<0.0001
Sputum *P. aeruginosa* density, log_{10} CFU/g, *n* (%)	7.4 ± 1.2	7.4 ± 1.3	7.4 ± 1.2	0.7577
High bacterial load	244 (68.3%)	108 (71.1%)	136 (66.3%)	0.6014
Moderate bacterial load	104 (29.1%)	40 (26.3%)	64 (31.2%)	–
Low bacterial load	9 (2.5%)	4 (2.6%)	5 (2.4%)	–
BSI score category, *n* (%)	–	–	–	0.0060
Mild	14 (3.9%%)	2 (1.3%)	12 (5.9%)	–
Moderate	73 (20.5%)	23 (15.1%)	50 (24.4%)	–
Severe	269 (75.4%)	126 (82.9%)	143 (69.8%)	–
Medications, *n* (%)	–	–	–	–
LABA	43 (12.0%)	29 (19.1%)	14 (6.8%)	0.0004
LAMA	59 (16.5%)	36 (23.7%)	23 (11.2%)	0.0017
ICS	26 (6.7%)	16 (10.5%)	10 (4.9%)	0.0423
Macrolides	33 (9.2%)	17 (11.2%)	16 (7.8%)	0.2757
Mucolytics	106 (29.7%)	48 (31.6%)	58 (28.3%)	0.5016

All values are mean ± SD or *n* (%) unless otherwise stated.
High-symptom burden: QoL-B-RSS<60; low-symptom burden: QoL-B-RSS≥60.
Mild severity: BSI 0–4; moderate severity: BSI 5–8; severe severity: BSI ≥9-point.
QoL-B-RSS: Quality-of-Life Bronchiectasis Respiratory Symptom Scale; BSI, bronchiectasis severity index; FEV_1, forced expiratory volume in one second; LABA, long-acting β2 agonists; LAMA, long-acting muscarinic receptor antagonists; ICS, inhaled corticosteroids; IQR, interquartile range.

relevant but distinct aspects of disease activity, and improvements in one domain may not translate directly into the changes in the other. Notably, florensocatib reduced sputum weight and purulence scores, particularly in the low-symptom-burden group, suggesting its biological activity on mucus production, which was not fully captured by the ordinal 1–4 patient-reported scale, necessitating more sensitive tools such as the visual analog scales in the future. In addition, a numerically minor improvement in the overall QoL-B-RSS score and several individual bronchitic items was observed in patients with lower baseline symptom burden, which was directionally consistent with the exacerbation reduction findings, although this did not reach statistical significance.

These findings highlight that antibiotics and anti-inflammatory treatments may preferentially target distinct aspects of bronchiectasis pathophysiology and underscore the importance of using multiple complementary endpoints when evaluating treatment effects in this heterogeneous disease. For instance, patients with lower symptom burden may preferentially benefit from anti-inflammatory drugs such as florensocatib for exacerbation prevention, whereas those with higher symptom burden, particularly in the presence of *Pseudomonas aeruginosa* infection, appear to gain greater symptomatic relief from inhaled antibiotics such as tobramycin. Moreover, in patients exhibiting pronounced inflammatory responses associated with *Pseudomonas* infections, combining DPP-1 inhibitors and inhaled antibiotics may offer additive benefits by simultaneously targeting distinct, but complementary, pathophysiological pathways. Prospective studies are warranted to validate these hypothesis-generating observations and to explore personalized and combination treatment approaches. Moreover, the observed stability of symptom burden subgroups in the placebo arms of

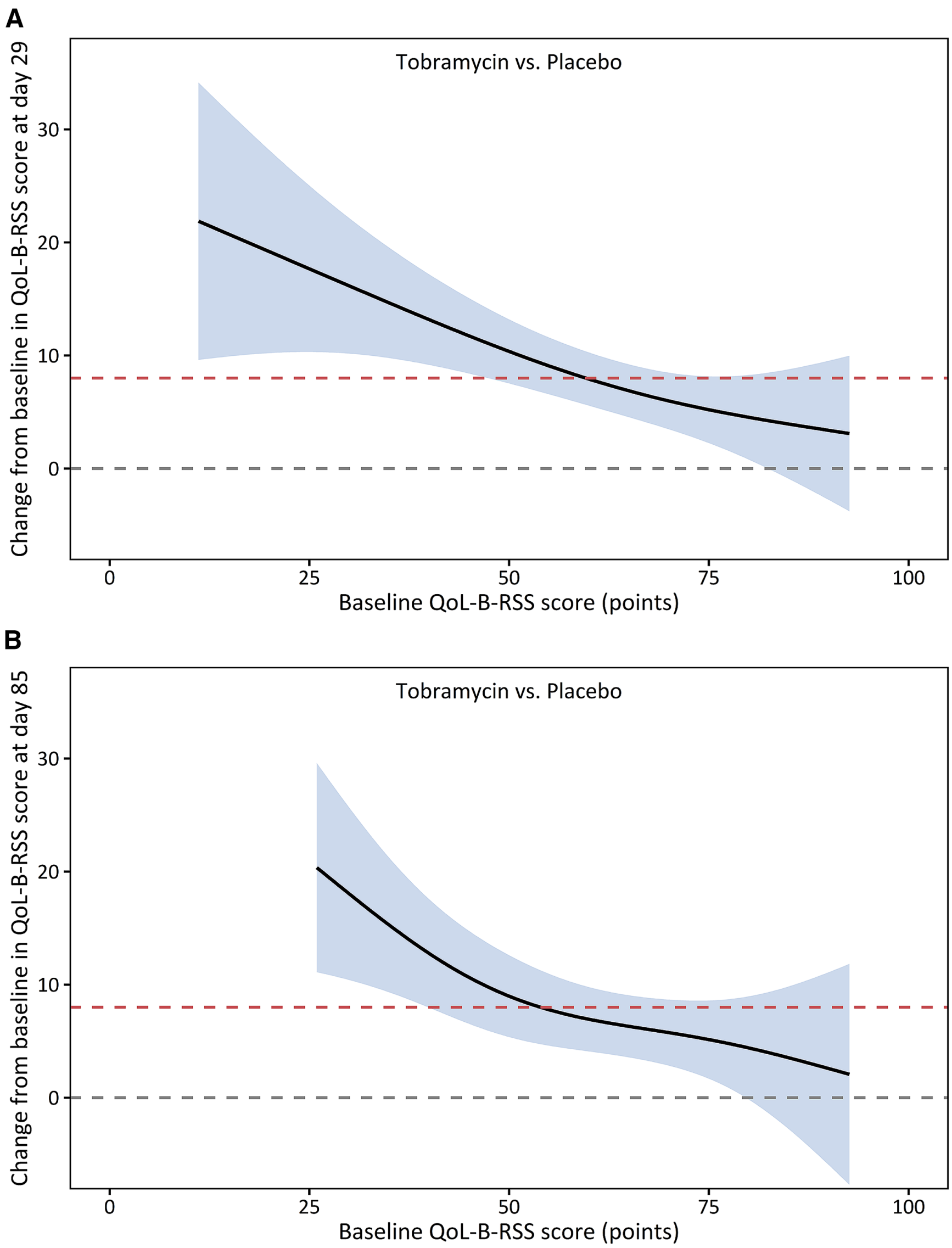

Figure 3. Association between baseline QoL-B-RSS score and the change of QoL-B-RSS score in the TORNASOL trial

Association between baseline QoL-B-RSS score and change in QoL-B-RSS score with inhaled tobramycin versus placebo in the TORNASOL trial. Panels show the mean difference in QoL-B-RSS change from baseline at (A) day 29 and (B) day 85. Solid lines represent the predicted mean difference derived from mixed-effects models, with shaded areas indicating 95% confidence intervals (CIs). The gray dashed line equaled to 0 denotes no treatment effect; the red dashed line equaled to 8 indicates the minimal clinically important difference (MCID). Values > 0 represent greater improvement of inhaled tobramycin versus control, whereas values ≥ 8 indicate clinically meaningful improvements.

both trials suggests that routine symptom assessment may capture a relatively stable clinical phenotype, which could have potential utility as a pragmatic marker to inform treatment response in bronchiectasis.[4,13,14]

In the TORNASOL trial, inhaled tobramycin produced statistically significant and clinically meaningful improvements in QoL-B-RSS scores (exceeding the MCID of 8 points in the high-symptom-burden group) and in bronchitic symptoms. These benefits are consistent with its anti-infective mechanism in a population where symptom burden is largely driven by chronic *Pseudomonas aeruginosa* infection.[12,23,24] Although some outcomes numerically favored the high-symptom-burden subgroup, the CIs overlapped substantially, indicating no clear statistical difference between the subgroups.

The two trials differed substantially in design, patient populations (including mandatory *Pseudomonas aeruginosa* infection in TORNASOL), follow-up duration, and endpoint definitions. These differences limit our direct cross-trial comparisons, and the observed patterns should be interpreted as hypothesis generating rather than confirmatory. We found no evidence that the clinically available markers of disease severity modified the association between baseline QoL-B-RSS and treatment outcomes, further cautioning against strong conclusions regarding symptom-based stratification.

This post hoc analysis suggests that baseline symptom burden may be associated with differential treatment responses in bronchiectasis—florensocatib with exacerbation reduction (numerically greater in lower-burden patients) and inhaled tobramycin with symptom improvement (greater in higher-burden patients). These hypothesis-generating findings warrant prospective validation in adequately powered trials to determine whether symptom burden can serve as a pragmatic stratification tool to guide personalized treatment.

Limitations of the study

Our study has limitations. First, in the post hoc analysis of two randomized controlled trials with different study designs and populations, the findings were exploratory and subject to potential bias. This difference limits direct comparison of short- versus long-term effects and should be considered when interpreting cross-trial patterns. Second, the use of a binary QoL-B-RSS cutoff (60 points), while practical, may result in a loss of information; continuous variable analyses were consistent with the main findings but showed no significant interactions. Third, in the TORNASOL trial, baseline QoL-B-RSS was used for both stratification and as an endpoint, introducing potential circularity for symptom-related outcomes. Although we have adjusted for the baseline QoL-B-RSS score in all models, the magnitude of symptomatic benefits

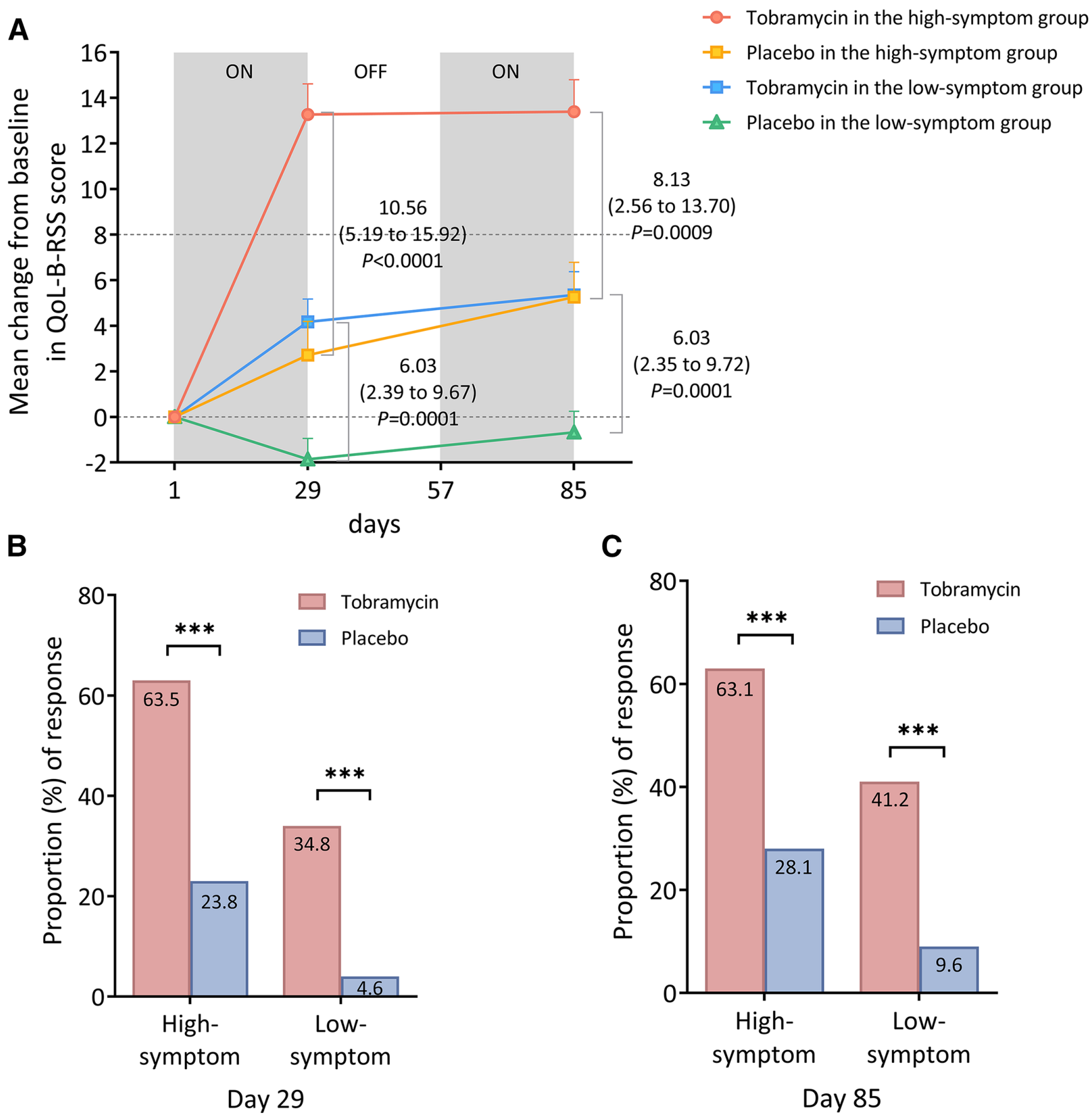

Figure 4. Changes in QoL-B-RSS from baseline and the proportion of patients achieving a clinically meaningful improvement in QoL-B-RSS in the TORNASOL trial

(A) Mean change from baseline for QoL-B-RSS in patients stratified by baseline symptom burden assessed by QoL-B-RSS and treatment arm. The red line denotes tobramycin treatment in the high-symptom group. The yellow line indicates placebo in the high-symptom group. The blue line represents tobramycin treatment in the low-symptom group. The green line corresponds to placebo in the low-symptom group. The gray shaded areas represent the on-treatment periods. The estimates have been adjusted for baseline symptom score. Time points: day 1 (baseline), day 29 (end of the first 28-day treatment cycle), day 57 (end of the first off-treatment period), day 85 (end of the second 28-day treatment cycle), and day 113 (1 month after the completion of all treatment). QoL-B-RSS data were not collected at day 57.

(B) Bar chart displays the percentage of patients with an improvement (increase) from baseline in QoL-B-RSS score of ≥8 points (established MCID threshold for the respiratory symptoms domain) at day 29. ***$p < 0.001$.

(C) Bar chart displays the percentage of patients with an improvement (increase) from baseline in QoL-B-RSS score of ≥8 points (established MCID threshold for the respiratory symptoms domain) at day 85. ***$p < 0.001$. High symptom burden: <60; low-symptom burden: ≥60. QoL-B-RSS, Quality-of-Life Bronchiectasis Respiratory Symptom Scale MCID, minimum clinically important difference.

in the stratified analyses should be interpreted with caution. Fourth, the limited sample size of the phase 2 SAVE-BE trial necessitated pooling of two florensocatib doses, potentially masking dose-specific effects. Also, the limited sample size might not be sufficient to fully power the post hoc analyses of lung function and quality of life. Currently, the phase 3 trial of florensocatib is ongoing, and therefore, our findings would be better validated when results are available. Fifth, our interaction analyses were partially restricted by the SAVE-BE trial where neither the BSI nor the bacterial colonization status at baseline was collected. Finally, informed by our domain-specific, longitudinal, and responder analyses, the composite nature of QoL-B-RSS may have limited its sensitivity to detect subtle or domain-specific effects of anti-inflammatory therapies such as florensocatib.

RESOURCE AVAILABILITY

Lead contact

Requests for further information and resources should be directed to and will be fulfilled by the lead contact, Professor Wei-Jie Guan (battery203@163.com).

Materials availability

This study did not generate new unique reagents.

Data and code availability

- This study does not generate new datasets. Data supporting the findings are available from the corresponding author upon reasonable request, subject to applicable data sharing policies of the original clinical trials.
- This paper does not report original code.
- Any additional information required to reanalyze the data reported in this paper is available from the lead contact upon request.

ACKNOWLEDGMENTS

The authors thank the Scientific Research Center of Guangzhou Medical University for providing instrument analysis and measurement. We thank Drs. Cang-Hong Zhi and Guan Wang (Joincare Pharmaceutical Co. Ltd.) for permission to use data from the TORNASOL phase 3 randomized trial and are grateful to the coordination led by Drs. Fang Jin and Juan Huang. Joincare Pharmaceutical Co. Ltd. played no role in data interpretation. We extend our appreciation to Ms. Dong Zhang and Xiao-Fei Wu (Haisco Co. Ltd.) for their efforts in conducting some of the post hoc analyses of the phase 2 SAVE-BE randomized trial of florensocatib. Funding: This work was supported by Noncommunicable Chronic Diseases - National Science and Technology Major Project (no. 2024ZD0529700 to Y.-H.G.), Major Project of Guangzhou National Laboratory (grant no. GZNL2024A02003 to W.-J.G.), Noncommunicable Chronic Diseases - National Science and Technology Major Project (no. 2024ZD0529600 to W.-J.G.), and National Natural Science Foundation of China (nos. 82270047 and 82570070 to Y.-H.G. and no. 82470040 to W.-J.G.).

AUTHOR CONTRIBUTIONS

Y-H.G., X-S.L., and W-J.G. contributed to the conception and design of the study. Y-H.G., X-S.L., X-L.S., F-Q.L., L-Y.S., H-M.L., and W-J.G. contributed to the interpretation of data. Y-H.G. and X-S.L. drafted the first version of the manuscript. All authors reviewed, edited, and approved the final manuscript.

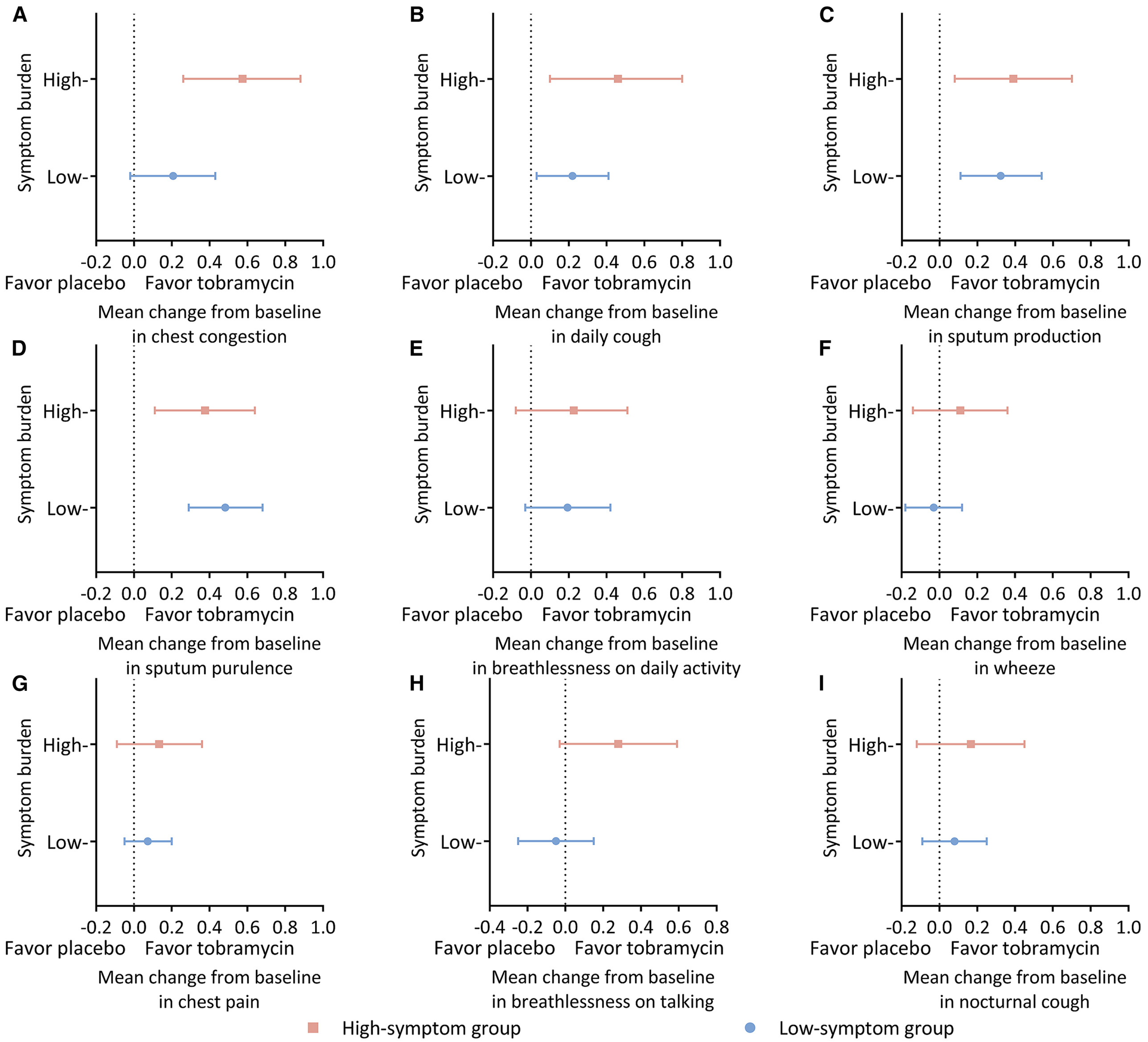

Figure 5. Mean changes from baseline in individual symptom domain scores of QoL-B-RSS during treatment, stratified by baseline symptom burden at day 29 in the TORNASOL trial

Forest plots of adjusted mean differences (tobramycin minus placebo) in the change from baseline for individual QoL-B-RSS symptom domain scores, in (A) chest congestion, (B) daily cough, (C) sputum production, (D) sputum purulence, (E) breathlessness on daily activity, (F) wheezing, (G) chest pain, (H) breathlessness on talking, and (I) nocturnal cough at day 29 (end of the first treatment cycle), stratified by baseline symptom burden assessed by QoL-B-RSS. Squares or dots represent the adjusted mean differences, and horizontal lines represent 95% CIs. Positive values favor tobramycin treatment; negative values favor placebo. Estimates are adjusted for baseline symptom score. High symptom burden: <60; low symptom burden: $\geq$60. QoL-B-RSS, Quality-of-Life Bronchiectasis Respiratory Symptom Scale; CI, confidence interval.

DECLARATION OF INTERESTS

The authors have no potential conflicts of interest to disclose.

DECLARATION OF GENERATIVE AI AND AI-ASSISTED TECHNOLOGIES IN THE WRITING PROCESS

There was no involvement of AI in preparation of this manuscript.

STAR★METHODS

Detailed methods are provided in the online version of this paper and include the following:

- KEY RESOURCES TABLE
- EXPERIMENTAL MODEL AND STUDY PARTICIPANT DETAILS
  - Study participants
- METHOD DETAILS

- Study design
- SAVE-BE trial procedures
- TORNASOL trial procedures
- Assessments and outcomes
- QUANTIFICATION AND STATISTICAL ANALYSIS
  - Additional resources

SUPPLEMENTAL INFORMATION

Supplemental information can be found online at https://doi.org/10.1016/j.xcrm.2026.102995.

Received: February 10, 2026
Revised: May 10, 2026
Accepted: July 22, 2026
Published: August 13, 2026

REFERENCES

1. Chalmers, J.D., Chang, A.B., Chotirmall, S.H., Dhar, R., and McShane, P.J. (2018). Bronchiectasis. Nat. Rev. Dis. Primers *4*, 45. https://doi.org/10.1038/s41572-018-0042-3.

2. Long, M.B., Chotirmall, S.H., Shteinberg, M., and Chalmers, J.D. (2024). Rethinking bronchiectasis as an inflammatory disease. Lancet Respir. Med. *12*, 901–914. https://doi.org/10.1016/S2213-2600(24)00176-0.

3. Chalmers, J.D., Aliberti, S., Filonenko, A., Shteinberg, M., Goeminne, P.C., Hill, A.T., Fardon, T.C., Obradovic, D., Gerlinger, C., Sotgiu, G., et al. (2018). Characterization of the "Frequent Exacerbator Phenotype" in Bronchiectasis. Am. J. Respir. Crit. Care Med. *197*, 1410–1420. https://doi.org/10.1164/rccm.201711-2202OC.

4. Gao, Y.H., Zheng, H.Z., Lu, H.W., Li, Y.Y., Feng, Y., Gu, S.Y., Sun, X.L., Mao, B., Bai, J.W., Liang, S., et al. (2024). Quality-of-Life Bronchiectasis Respiratory Symptom Scale predicts the risk of exacerbations in adults with bronchiectasis: a prospective observational study. Ann. Am. Thorac. Soc. *21*, 393–401. https://doi.org/10.1513/AnnalsATS.202302-133OC.

5. Hill, A.T., Sullivan, A.L., Chalmers, J.D., De Soyza, A., Elborn, S.J., Floto, A.R., Grillo, L., Gruffydd-Jones, K., Harvey, A., Haworth, C.S., et al. (2019). British Thoracic Society Guideline for bronchiectasis in adults. Thorax *74*, 1–69. https://doi.org/10.1136/thoraxjnl-2018-212463.

6. Chalmers, J.D., Haworth, C.S., Flume, P., Long, M.B., Burgel, P.R., Dimakou, K., Blasi, F., Herrero-Cortina, B., Dhar, R., Chotirmall, S.H., et al. (2025). European Respiratory Society Clinical Practice Guideline for the Management of Adult Bronchiectasis. Eur. Respir. J. *66*, 2501126. https://doi.org/10.1183/13993003.01126-2025.

7. Chalmers, J.D., Boersma, W., Lonergan, M., Jayaram, L., Crichton, M.L., Karalus, N., Taylor, S.L., Martin, M.L., Burr, L.D., Wong, C., and Altenburg, J. (2019). Long-term macrolide antibiotics for the treatment of bronchiectasis in adults: an individual participant data meta-analysis. Lancet Respir. Med. *7*, 845–854. https://doi.org/10.1016/S2213-2600(19)30191-2.

8. Cordeiro, R., Choi, H., Haworth, C.S., and Chalmers, J.D. (2024). The efficacy and safety of inhaled antibiotics for the treatment of bronchiectasis in adults: updated systematic review and meta-analysis. Chest *166*, 61–80. https://doi.org/10.1016/j.chest.2024.01.045.

9. Guan, W.J., Xu, J.F., Luo, H., Xu, X.X., Song, Y.L., Ma, W.L., Liang, Z.A., Liu, X.D., Zhang, G.J., Zhang, X.J., et al. (2023). A double-blind randomized placebo-controlled phase 3 trial of tobramycin inhalation solution in adults with bronchiectasis with Pseudomonas aeruginosa infection. Chest *163*, 64–76. https://doi.org/10.1016/j.chest.2022.07.007.

10. Crichton, M.L., Lonergan, M., Barker, A.F., Sibila, O., Goeminne, P., Shoemark, A., and Chalmers, J.D. (2020). Inhaled aztreonam improves symptoms of cough and sputum production in patients with bronchiectasis: a post hoc analysis of the AIR-BX studies. Eur. Respir. J. *56*, 2000608. https://doi.org/10.1183/13993003.00608-2020.

11. Sibila, O., Laserna, E., Shoemark, A., Keir, H.R., Finch, S., Rodrigo-Troyano, A., Perea, L., Lonergan, M., Goeminne, P.C., and Chalmers, J.D. (2019). Airway bacterial load and inhaled antibiotic response in bronchiectasis. Am. J. Respir. Crit. Care Med. *200*, 33–41. https://doi.org/10.1164/rccm.201809-1651OC.

12. Chalmers, J.D., Cipolla, D., Thompson, B., Davis, A.M., O'Donnell, A., Tino, G., Gonda, I., Haworth, C., and Froehlich, J. (2020). Changes in respiratory symptoms during 48-week treatment with ARD-3150 (inhaled liposomal ciprofloxacin) in bronchiectasis: results from the ORBIT-3 and-4 studies. Eur. Respir. J. *56*, 2000110. https://doi.org/10.1183/13993003.00110-2020.

13. Sibila, O., Stobo, J., Perea, L., Gao, Y.H., Xu, J.F., Lind, H., Viligorska, K., Spinou, A., Polverino, E., Ringshausen, F.C., et al. (2025). Symptoms, risk of future exacerbations, and response to long-term macrolide treatment in bronchiectasis: an observational study. Lancet Respir. Med. *13*, 911–920. https://doi.org/10.1016/S2213-2600(25)00160-2.

14. Gao, Y.H., Abo Leyah, H., Finch, S., Lonergan, M., Aliberti, S., De Soyza, A., Fardon, T.C., Tino, G., and Chalmers, J.D. (2020). Relationship between symptoms, exacerbations, and treatment response in bronchiectasis. Am. J. Respir. Crit. Care Med. *201*, 1499–1507. https://doi.org/10.1164/rccm.201910-1972OC.

15. Chalmers, J.D., Metersky, M., Aliberti, S., Morgan, L., Fucile, S., Lauterio, M., and McDonald, P.P. (2025). Neutrophilic inflammation in bronchiectasis. Eur. Respir. Rev. *34*, 240179. https://doi.org/10.1183/16000617.0179-2024.

16. Chalmers, J.D., Mall, M.A., Chotirmall, S.H., O'Donnell, A.E., Flume, P.A., Hasegawa, N., Ringshausen, F.C., Watz, H., Xu, J.F., Shteinberg, M., and McShane, P.J. (2025). Targeting neutrophil serine proteases in bronchiectasis. Eur. Respir. J. *65*, 2401050. https://doi.org/10.1183/13993003.01050-2024.

17. Chalmers, J.D., Haworth, C.S., Metersky, M.L., Loebinger, M.R., Blasi, F., Sibila, O., O'Donnell, A.E., Sullivan, E.J., Mange, K.C., Fernandez, C., et al. (2020). Phase 2 Trial of the DPP-1 Inhibitor Brensocatib in Bronchiectasis. N. Engl. J. Med. *383*, 2127–2137. https://doi.org/10.1056/NEJMoa2021713.

18. Cipolla, D., Zhang, J., Korkmaz, B., Chalmers, J.D., Basso, J., Lasala, D., Fernandez, C., Teper, A., Mange, K.C., Perkins, W.R., and Sullivan, E.J. (2023). Dipeptidyl peptidase-1 inhibition with brensocatib reduces the activity of all major neutrophil serine proteases in patients with bronchiectasis: results from the WILLOW trial. Respir. Res. *24*, 133. https://doi.org/10.1186/s12931-023-02444-z.

19. Johnson, E.D., Long, M.B., Perea, L., Shih, V.H., Fernandez, C., Teper, A., Cipolla, D., McIntosh, E., Galloway, R., Eke, Z., et al. (2025). Broad Immunomodulatory Effects of the Dipeptidyl Peptidase-1 Inhibitor Brensocatib in Bronchiectasis: Data from the Phase 2, Double-Blind, Placebo-controlled WILLOW Trial. Am. J. Respir. Crit. Care Med. *211*, 770–778. https://doi.org/10.1164/rccm.202408-1545OC.

20. Zhong, N.S., Qiu, R., Cao, J., Huang, Y.M., Zhou, H., Xu, X.X., Xu, J.F., Ye, H., Yang, Z.R., Gao, L.Y., et al. (2025). Effects of the DPP-1 inhibitor HSK31858 in adults with bronchiectasis in China (SAVE-BE): a phase 2, multicentre, double-blind, randomised, placebo-controlled trial. Lancet Respir. Med. *13*, 414–424. https://doi.org/10.1016/S2213-2600(25)00019-0.

21. Chalmers, J.D., Burgel, P.R., Daley, C.L., De Soyza, A., Haworth, C.S., Mauger, D., Loebinger, M.R., McShane, P.J., Ringshausen, F.C., Blasi, F., et al. (2025). Phase 3 Trial of the DPP-1 Inhibitor Brensocatib in Bronchiectasis. N. Engl. J. Med. *392*, 1569–1581. https://doi.org/10.1056/NEJMoa2411664.

22. Quittner, A.L., O'Donnell, A.E., Salathe, M.A., Lewis, S.A., Li, X., Montgomery, A.B., O'Riordan, T.G., and Barker, A.F. (2015). Quality of Life Questionnaire-Bronchiectasis: final psychometric analyses and determination of minimal important difference scores. Thorax *70*, 12–20. https://doi.org/10.1136/thoraxjnl-2014-205918.

23. Finch, S., McDonnell, M.J., Abo-Leyah, H., Aliberti, S., and Chalmers, J.D. (2015). A comprehensive analysis of the impact of Pseudomonas aeruginosa

colonization on prognosis in adult bronchiectasis. Ann. Am. Thorac. Soc. *12*, 1602–1611. https://doi.org/10.1513/AnnalsATS.201506-333OC.

24. Araugo, D., Shteinberg, M., and Aliberti, S. (2018). The independent contribution of Pseudomonas aeruginosa infection to long-term clinical outcomes in bronchiectasis. Eur. Respir. J. *51*, 1701953.

25. Hill, A.T., Haworth, C.S., Aliberti, S., Barker, A., Blasi, F., Boersma, W., Chalmers, J.D., De Soyza, A., Dimakou, K., Elborn, J.S., et al. (2017). Pulmonary exacerbation in adults with bronchiectasis: a consensus definition for clinical research. Eur. Respir. J. *49*, 1700051. https://doi.org/10.1183/13993003.00051-2017.

CellPress
OPEN ACCESS

STAR★METHODS

KEY RESOURCES TABLE

REAGENT or RESOURCE	SOURCE	IDENTIFIER
Deposited data		
Patients' data of SAVE-BE clinical trial	Parent SAVE-BE clinical trial dataset	ClinicalTrials.gov: NCT05601778
Patients' data of TORNASOL clinical trial	Parent TORNASOL clinical trial dataset	ClinicalTrials.gov: NCT03715322
Software and algorithms		
SPSS (version 22.0)	IBM	https://www.ibm.com/spss
SAS (version 9.4)	SAS Institute	https://www.sas.com/
R (version 4.1.1)	R Foundation	https://www.r-project.org/
mgcv (R package)	Wood SN.	https://cran.r-project.org/package=mgcv
Other		
Quality of Life-Bronchiectasis Respiratory Symptom Scale (QoL-B-RSS)	Quittner AL et al.	https://doi.org/10.1136/thoraxjnl-2014-205918

EXPERIMENTAL MODEL AND STUDY PARTICIPANT DETAILS

Study participants

This is a *post-hoc* analysis of two multicenter, randomized, double-blind, placebo-controlled trials conducted in China: SAVE-BE (NCT05601778) and TORNASOL (NCT03715322).

The SAVE-BE trial included 224 adult outpatients aged ≥18 years with HRCT-confirmed clinically significant bronchiectasis, ≥2 exacerbations in the prior 12 months, body-mass index (BMI) ≥18 kg/m^2, and daily spontaneous sputum production.[20] The TORNASOL trial enrolled 357 patients aged 18 to 75 years with symptomatic bronchiectasis confirmed by HRCT and *Pseudomonas aeruginosa* isolation and one or more exacerbations in the prior two years. Baseline characteristics in the TORNASOL trial were reported for the full randomized population (n = 357), while outcome analyses used the modified intention-to-treat (mITT) population (n = 339), consistent with the parent trial.[9]

Details of the demographics of the patients included in these two clinical trials are shown in Tables 1 and 2. Both trials were approved by the institutional ethics committees of all participating centers and were conducted in accordance with the Declaration of Helsinki and Good Clinical Practice guidelines. All participants provided written informed consent prior to enrollment.

METHOD DETAILS

Study design

This was a *post hoc* and exploratory analysis of the SAVE-BE and TORNASOL trials, aiming to evaluate whether baseline symptom burden was associated with treatment responses.

SAVE-BE trial procedures

The SAVE-BE trial, a phase 2, multicenter, randomized, double-blind, placebo-controlled study, was conducted at 25 tertiary centers in China. Participants were randomly assigned (1:1:1) to receive 20 mg or 40 mg florensocatib or placebo, with stratification by the exacerbation frequency in the previous year (<3 vs. ≥ 3). Treatment allocation was concealed, and participants and investigators were blinded to group assignment. Patients underwent a 4-week screening period, a 24-week treatment period, and a 4-week follow-up. the primary endpoint was the annualized exacerbation frequency. Secondary endpoints included the time to the first exacerbation, the proportion of exacerbation-free patients, and the change from baseline in FEV_1% predicted, 24-h sputum weight, and sputum purulence score (8-point scale) up to 24 weeks.

TORNASOL trial procedures

The TORNASOL trial, a phase 3, multicenter, randomized, double-blind, placebo-controlled study in China, randomly assigned eligible participants (1:1) to receive nebulized tobramycin (300 mg twice daily) or placebo (5 mL saline twice daily) in two 28-day on/off cycles (totaling 16 weeks), with follow-up to day 85. Randomization was performed using a computer-generated permuted block sequence with stratification by center. Treatment allocation was concealed, and both participants and investigators were blinded to group assignment. In TORNASOL, the co-primary endpoints were the change from baseline in *Pseudomonas aeruginosa* sputum density and QoL-B-RSS score at day 29.

Assessments and outcomes

The QoL-B-RSS, a validated tool, assessed respiratory symptoms (Table S7) on a 0–100 point scale (lower scores indicate greater severity; MCID, 8 points).[22] Patients were stratified into high (QoL-B-RSS <60) or low ($\geq$60) symptom burden groups based on the median baseline score in SAVE-BE (62.96) and TORNASOL (62.96), rounded to 60 for ease in clinical practice.[6,13]

Exacerbations were defined per the EMBARC criteria: at least 3 symptoms (e.g., cough, sputum purulence, dyspnea) for longer than 48 h requiring antibiotics, with severe exacerbations necessitating hospitalization.[25]

In SAVE-BE, the *post-hoc* study outcomes included the exacerbation frequency, the time to the first exacerbation, and the changes in QoL-B-RSS up to 24 weeks. In TORNASOL, the *post-hoc* study outcomes were QoL-B-RSS change at day 29, with follow-up to day 85. Individual QoL-B-RSS items were scored from 1 (most severe) to 4 (least severe), with sputum purulence responses adjusted as per the published study.[22]

QUANTIFICATION AND STATISTICAL ANALYSIS

These analyses were exploratory and *post-hoc* in nature. We conducted analyses using IBM SPSS Statistics v22.0, SAS v9.4, and R (version 4.1.1; mgcv package). Data were reported as numbers (%) for categorical variables and as means (SD), or medians (IQR) for continuous variables, as appropriate. Between-group comparisons adopted the t-tests or analysis-of-variance for continuous variables, chi-square or Fisher's exact tests for categorical variables, and nonparametric equivalents as needed. All statistical tests were two-sided, and a p value < 0.05 was considered statistically significant. Detailed statistical results, including exact p values, effect estimates, and confidence intervals (CI), are reported in the results section and corresponding figure legends or tables.

In the SAVE-BE trial, generalized additive models (GAMs) with negative binomial distribution (exacerbation frequency) or Cox regression models (the time to the first exacerbation) were adopted to assess the impacts of baseline QoL-B-RSS score on the treatment effects for these outcomes. Analysis of covariance was employed to evaluate the changes from baseline in FEV_1, 24-h sputum weight, sputum purulence scores and QoL-B-RSS, with their corresponding baseline levels and exacerbation frequency within one year before enrollment (cutoff: three events) included as covariates. The two florensocatib doses (20 mg and 40 mg) were pooled to increase the statistical power, while noting that the 40 mg dose demonstrated numerically greater efficacy than the 20 mg dose in the parent trial.

In the TORNASOL trial, GAM was adopted to assess the impacts of baseline QoL-B-RSS score on the treatment effects of the change from baseline in QoL-B-RSS. Mixed-effects models were used to evaluate QoL-B-RSS changes, including the treatment, time, baseline symptoms as fixed effects, with random effects for baseline variability. Bonferroni correction was adopted to adjust for multiple comparisons.

Exploratory analyses for the primary endpoints were conducted with stratification by baseline FEV_1% predicted (<50% vs. $\geq$ 50%) and the prior exacerbation history (2 vs. $\geq$ 3 events) in the SAVE-BE trial, and by Bronchiectasis Severity Index (BSI) (0–8 vs. $\geq$ 9) in the TORNASOL trial, with formal interaction testing.

The number of participants (n) for each analysis population is provided in the corresponding tables and represents the number of patients included in that specific analysis. Inclusion and exclusion of participants were based on the predefined criteria of the parent trials, and no additional exclusion criteria were applied for the present *post hoc* analyses. No formal sample size calculations were performed for these *post-hoc* exploratory analyses and sample sizes were determined in the parent trials.[9,20]

Additional resources

These two parent clinical trials have been registered with ClinicalTrials.gov NCT05601778 and NCT03715322.

Cell Reports Medicine, Volume *7*

Supplemental information

Symptoms and treatment response to florensocatib and inhaled tobramycin in bronchiectasis: Post hoc analysis of two randomized trials

Yong-Hua Gao (高永华), Xue-Shan Li (李雪杉), Xiao-Li Sun (孙晓丽), Fang-Qiong Li (李芳琼), Lu-Yin Sun (孙露银), Han-Mo Liu (刘翰墨), and Wei-Jie Guan (关伟杰)

Supplemental information

Symptoms and Treatment Response to DPP-1 Inhibitor Florensocatib and Inhaled Tobramycin in Adults with Bronchiectasis: *Post-hoc* Analysis of Two Multicenter Randomized, Placebo-Controlled Trials

Yong-Hua Gao, Xue-Shan Li, Xiao-Li Sun, Fang-Qiong Li, Lu-Yin Sun, Han-Mo Liu, Wei-Jie Guan.

Supplementary tables

Table S1. Baseline symptom burden assessed by QoL-B-RSS of patients with bronchiectasis in SAVE-BE and TORNASOL trial. Related to Table 1 and Table 2.

Symptom burden groups	SAVE-BE trial				TORNASOL trial			
	Total (N=224)	Florensocatib (n=149)	Placebo (n=75)	*P*-value	Total (N=357)	Tobramycin (n=178)	Placebo (n=179)	*P*-value
High-symptom group, n (%)	107 (47.8%)	71 (47.7%)	36 (48.0%)	0.961	152 (42.6%)	84 (47.2%)	68 (38.0%)	0.079
Low-symptom group, n (%)	117 (52.2%)	78 (52.3%)	39 (52.0%)		205 (57.4%)	94 (52.8%)	111 (62.0%)	

All values are n (%). High-symptom burden: QoL-B-RSS<60; low-symptom burden: QoL-B-RSS≥60. Definition of abbreviations: QoL-B-RSS=Quality-of-Life Bronchiectasis Respiratory Symptoms Scale.

Table S2. Sputum characteristics, lung function and respiratory symptoms at week 24 stratified by different symptom burden subgroups in the SAVE-BE trial. Related to Figure 1 and Figure 2.

Endpoints and subgroups	Florensocatib		Placebo		Mean difference versus placebo (95%CI)	*P*-value
	No.	LSM±SE	No.	LSM±SE		
**24-hour sputum weight, g**						
All patients in SAVE-BE trial	132	-8.13±1.83	64	0.29±2.50	-8.43 (-14.00 to -2.85)	0.0032
High-symptom group	60	-14.20±3.65	31	-3.32±4.60	-10.89 (-21.46 to -0.31)	0.0437
Low-symptom group	72	-2.89±1.61	33	2.88±2.34	-5.77 (-10.93 to -0.62)	0.0286
**Sputum purulence score, points**						
All patients in SAVE-BE trial	132	-0.70±0.17	64	0.10±0.23	-0.80 (-1.28 to -0.26)	0.0031
High-symptom group	60	-0.70±0.25	31	0.00±0.31	-0.70 (-1.38 to 0.04)	0.0653
Low-symptom group	72	-0.60±0.23	33	0.30±0.33	-0.90 (-1.64 to -0.20)	0.0133
**FEV_1 percentage predicted, %**						
All patients in SAVE-BE trial	133	-1.71±0.68	63	-2.43±0.93	0.72 (-1.33 to 2.77)	0.4896
High-symptom group	61	-1.81±1.03	31	-1.38±1.28	-0.43 (-3.37 to 2.51)	0.7727
Low-symptom group	72	-1.70±0.93	32	-3.46±1.37	1.76 (-1.23 to 4.74)	0.2457
**QoL-B-RSS, points**						
All patients in SAVE-BE trial	133	5.73±1.26	64	3.90±1.68	1.83 (-1.93 to 5.60)	0.3380
High-symptom group	61	8.55±2.13	31	11.30±2.59	-2.75 (-8.91 to 3.41)	0.3770
Low-symptom group	72	2.20±1.48	33	-2.33±2.13	4.53 (-0.19 to 9.25)	0.0598

The LSE±SE of different treatment represents the changes from baseline to week 24. All estimates have been adjusted with the baseline levels and the exacerbation frequency within the previous year (cut-off: 3 events). High-symptom group: QoL-B-RSS<60; low-symptom group: QoL-B-RSS≥60. QoL-B-RSS: Quality-of-Life of Bronchiectasis Respiratory Symptom Scale; FEV_1: forced expiratory volume in one second; LSM: least-square mean; SE: standard error; CI: confidence interval.

Table S3. Mean changes from baseline in individual respiratory symptoms of QoL-B-RSS in patients receiving florensocatib versus placebo in the SAVE-BE trial. Related to Figure 1 and Figure 2.

Items	Visits	Florensocatib		Placebo (Mean±SE)		Mean Difference vs placebo (95%CI)	*P*-value
		No.	Mean±SE	No.	Mean±SE		
Congestion							
	Week 4	144	0.19±0.98	72	0.24±0.97	-0.06 (-0.28- 0.16)	0.6071
	Week 12	137	0.32±1.13	66	0.24±1.19	0.10 (-0.15- 0.36)	0.4358
	Week 20	133	0.32±1.00	64	0.28±1.13	0.07 (-0.17- 0.31)	0.5602
	Week 24	133	0.34±0.99	64	0.33±1.04	0.06 (-0.18- 0.30)	0.6061
Daily cough							
	Week 4	144	0.05±0.80	72	0.08±0.92	0.00 (-0.21- 0.21)	0.9998
	Week 12	137	0.30±0.81	66	0.24±0.79	0.11 (-0.10- 0.32)	0.3090
	Week 20	133	0.31±0.83	64	0.30±0.85	0.08 (-0.12- 0.29)	0.4265
	Week 24	133	0.28±0.76	64	0.20±0.78	0.14 (-0.06- 0.34)	0.1594
Sputum production							
	Week 4	144	0.09±0.98	72	0.32±0.98	-0.16 (-0.41- 0.08)	0.1935
	Week 12	137	0.28±0.95	66	0.30±0.91	0.07 (-0.19- 0.32)	0.6016
	Week 20	133	0.27±0.90	64	0.36±0.95	-0.01 (-0.25- 0.23)	0.9562
	Week 24	133	0.34±0.89	64	0.31±0.83	0.09 (-0.14- 0.33)	0.4299
Sputum purulence							
	Week 4	144	-0.02±0.72	72	-0.06±0.65	0.02 (-0.16- 0.20)	0.8255
	Week 12	137	0.01±0.69	66	-0.11±0.73	0.09 (-0.10- 0.27)	0.3628
	Week 20	133	0.09±0.70	64	-0.16±0.70	0.24 (0.05- 0.44)	0.0140
	Week 24	133	-0.02±0.74	64	-0.13±0.71	0.09 (-0.11- 0.29)	0.3842
Breathlessness on daily activity							
	Week 4	144	0.10±0.81	72	0.13±0.77	0.03 (-0.17- 0.23)	0.7539
	Week 12	137	0.18±0.85	66	0.14±0.93	0.13 (-0.09- 0.34)	0.2533
	Week 20	133	0.24±0.87	64	0.23±0.87	0.09 (-0.13- 0.30)	0.4240
	Week 24	133	0.21±0.90	64	0.17±0.77	0.11 (-0.11- 0.33)	0.3210
Wheeze							
	Week 4	144	0.10±0.72	72	0.15±0.87	0.03 (-0.16- 0.22)	0.7608
	Week 12	137	0.23±0.83	66	0.18±0.98	0.15 (-0.05- 0.35)	0.1394
	Week 20	133	0.22±0.71	64	0.23±0.83	0.07 (-0.12- 0.26)	0.4556

	Week 24	133	0.20±0.69	64	0.28±0.77	0.01 (-0.16- 0.18)	0.9036
Chest pain							
	Week 4	144	0.01±0.55	72	0.03±0.73	-0.01 (-0.16- 0.15)	0.9240
	Week 12	137	0.17±0.68	66	0.15±0.79	0.03 (-0.13- 0.20)	0.6997
	Week 20	133	0.19±0.59	64	0.16±0.72	0.05 (-0.10- 0.19)	0.5270
	Week 24	133	0.15±0.63	64	0.08±0.67	0.09 (-0.06- 0.23)	0.2525
Breathlessness on talking							
	Week 4	144	0.02±0.61	72	0.17±0.80	-0.05 (-0.23- 0.13)	0.5782
	Week 12	137	0.08±0.63	66	0.17±0.81	0.01 (-0.17- 0.20)	0.8909
	Week 20	133	0.02±0.63	64	0.19±0.83	-0.04 (-0.23- 0.15)	0.6932
	Week 24	133	0.02±0.72	64	0.16±0.70	0.00 (-0.19- 0.19)	0.9986
Nocturnal cough							
	Week 4	144	0.10±0.62	72	0.07±0.66	0.11 (-0.05- 0.26)	0.1728
	Week 12	137	0.20±0.63	66	0.14±0.78	0.14 (-0.03- 0.30)	0.1125
	Week 20	133	0.20±0.67	64	0.14±0.83	0.17 (-0.01- 0.35)	0.0587
	Week 24	133	0.08±0.62	64	0.11±0.80	0.07 (-0.10- 0.24)	0.4358

Mean changes from baseline in individual QoL-B-RSS symptom domains between florensocatib and placebo. Abbreviations: QoL-B-RSS=Quality-of-Life Bronchiectasis Respiratory Symptoms Scale; SE: standard error; CI: confidence interval.

Table S4. Mean changes from baseline in individual respiratory symptoms of QoL-B-RSS in patients with high symptom burden receiving florensocatib versus placebo in the SAVE-BE trial. Related to Figure 1 and Figure 2.

Items	Visits	Florensocatib		Placebo (Mean±SE)		Mean Difference vs placebo (95%CI)	*P*-value
		No.	Mean±SE	No.	Mean±SE		
Congestion							
	Week 4	68	0.50±0.87	35	0.57±0.92	-0.06 (-0.38- 0.26)	0.7199
	Week 12	64	0.67±1.05	32	0.75±1.16	-0.04 (-0.44- 0.37)	0.8624
	Week 20	61	0.61±1.04	30	0.87±1.01	-0.16 (-0.57- 0.24)	0.4318
	Week 24	61	0.62±0.93	31	0.81±1.01	-0.13 (-0.50- 0.25)	0.5082
Daily cough							
	Week 4	68	0.18±0.90	35	0.23±1.03	0.00 (-0.33- 0.32)	0.9890
	Week 12	64	0.48±0.94	32	0.50±0.88	0.03 (-0.34- 0.39)	0.8876
	Week 20	61	0.49±0.89	30	0.63±0.76	-0.08 (-0.42- 0.26)	0.6467
	Week 24	61	0.39±0.86	31	0.45±0.81	-0.01 (-0.35- 0.32)	0.9324
Sputum production							
	Week 4	68	0.25±1.03	35	0.63±0.94	-0.23 (-0.61- 0.15)	0.2338
	Week 12	64	0.45±0.89	32	0.66±0.87	-0.12 (-0.50- 0.26)	0.5417
	Week 20	61	0.41±0.90	30	0.70±0.95	-0.13 (-0.52- 0.25)	0.5005
	Week 24	61	0.48±0.83	31	0.61±0.92	-0.03 (-0.39- 0.33)	0.8721
Sputum purulence							
	Week 4	68	0.02±0.75	35	0.06±0.59	-0.01 (-0.27- 0.24)	0.9097
	Week 12	64	0.00±0.74	32	0.00±0.72	0.03 (-0.24- 0.29)	0.8451
	Week 20	61	0.14±0.63	30	-0.10±0.66	0.29 (0.04- 0.55)	0.0239
	Week 24	61	0.05±0.65	31	-0.03±0.61	0.09 (-0.18- 0.35)	0.5136
Breathlessness on daily activity							
	Week 4	68	0.25±0.92	35	0.09±0.92	-0.01 (-0.35- 0.32)	0.9315
	Week 12	64	0.36±0.82	32	0.25±0.95	-0.02 (-0.35- 0.32)	0.9249
	Week 20	61	0.39±0.90	30	0.30±0.84	-0.03 (-0.38- 0.31)	0.8414
	Week 24	61	0.36±0.91	31	0.26±0.73	-0.03 (-0.36- 0.31)	0.8667
Wheeze							
	Week 4	68	0.22±0.91	35	0.26±1.01	0.01 (-0.32- 0.34)	0.9367
	Week 12	64	0.59±0.92	32	0.19±0.93	0.31 (-0.02- 0.64)	0.0628
	Week 20	61	0.39±0.88	30	0.33±0.99	-0.07 (-0.41- 0.27)	0.6808

	Week 24	61	0.43±0.83	31	0.29±0.82	-0.11 (-0.40- 0.19)	0.4766
Chest pain							
	Week 4	68	0.04±0.53	35	0.09±0.92	-0.02 (-0.26- 0.22)	0.8707
	Week 12	64	0.28±0.79	32	0.25±0.95	0.07 (-0.20- 0.35)	0.5934
	Week 20	61	0.28±0.71	30	0.30±0.84	0.01 (-0.23- 0.26)	0.9168
	Week 24	61	0.26±0.73	31	0.26±0.73	0.02 (-0.22- 0.26)	0.8693
Breathlessness on talking							
	Week 4	68	0.01±0.82	35	0.26±1.01	-0.11 (-0.44- 0.22)	0.5162
	Week 12	64	0.16±0.78	32	0.19±0.93	0.06 (-0.26- 0.38)	0.7115
	Week 20	61	0.11±0.75	30	0.33±0.99	-0.07 (-0.40- 0.27)	0.6807
	Week 24	61	0.11±0.80	31	0.29±0.82	-0.03 (-0.35- 0.29)	0.8390
Nocturnal cough							
	Week 4	68	0.19±0.65	35	0.29±0.71	0.01 (-0.23- 0.26)	0.9138
	Week 12	64	0.31±0.73	32	0.25±0.95	0.16 (-0.13- 0.46)	0.2713
	Week 20	61	0.39±0.71	30	0.33±0.92	0.29 (0.01- 0.57)	0.0453
	Week 24	61	0.20±0.60	31	0.23±0.88	0.15 (-0.10- 0.39)	0.2432

Mean changes from baseline in individual QoL-B-RSS symptom domains between florensocatib and placebo in patient with high symptom burden (QoL-B-RSS<60).

Abbreviations: QoL-B-RSS=Quality-of-Life Bronchiectasis Respiratory Symptoms Scale; SE: standard error; CI: confidence interval.

Table S5. Mean changes from baseline in individual respiratory symptoms of QoL-B-RSS in patients with low symptom burden receiving florensocatib versus placebo in the SAVE-BE trial. Related to Figure 1 and Figure 2.

**Items**	**Visits**	**Florensocatib**		**Placebo (Mean±SE)**		**Mean Difference vs placebo (95%CI)**	***P*-value**
		**No.**	**Mean±SE**	**No.**	**Mean±SE**		
Congestion							
	Week 4	76	-0.08±0.99	37	-0.08±0.92	-0.09 (-0.41- 0.23)	0.5693
	Week 12	73	0.01±1.11	34	-0.24±1.02	0.17 (-0.15- 0.49)	0.2901
	Week 20	72	0.08±0.92	34	-0.24±0.99	0.26 (-0.02- 0.55)	0.0695
	Week 24	72	0.10±0.98	33	-0.12±0.86	0.20 (-0.12- 0.51)	0.2160
Daily cough							
	Week 4	76	-0.07±0.68	37	-0.05±0.78	-0.01 (-0.29- 0.26)	0.9192
	Week 12	73	0.14±0.63	34	0.00±0.60	0.16 (-0.08- 0.39)	0.1860
	Week 20	72	0.15±0.74	34	0.00±0.82	0.23 (-0.02- 0.48)	0.0676
	Week 24	72	0.18±0.66	33	-0.03±0.68	0.29 (0.05- 0.53)	0.0199
Sputum production							
	Week 4	76	-0.05±0.92	37	0.03±0.93	-0.10 (-0.43- 0.23)	0.5454
	Week 12	73	0.14±0.99	34	-0.03±0.83	0.17 (-0.17- 0.52)	0.3268
	Week 20	72	0.15±0.88	34	0.06±0.85	0.09 (-0.22- 0.40)	0.5655
	Week 24	72	0.22±0.92	33	0.03±0.64	0.18 (-0.13- 0.50)	0.2521
Sputum purulence							
	Week 4	76	-0.05±0.68	37	-0.17±0.70	0.06 (-0.19- 0.32)	0.6106
	Week 12	73	0.01±0.64	34	-0.21±0.74	0.15 (-0.11- 0.42)	0.2575
	Week 20	72	0.06±0.75	34	-0.21±0.74	0.20 (-0.09- 0.50)	0.1758
	Week 24	72	-0.09±0.81	33	-0.22±0.79	0.04 (-0.26- 0.35)	0.7818
Breathlessness on daily activity							
	Week 4	76	-0.03±0.67	37	-0.16±0.55	0.11 (-0.12- 0.35)	0.3351
	Week 12	73	0.03±0.85	34	-0.26±0.71	0.26 (-0.03- 0.56)	0.0805
	Week 20	72	0.11±0.83	34	-0.15±0.66	0.21 (-0.06- 0.49)	0.1275
	Week 24	72	0.08±0.87	33	-0.21±0.55	0.23 (-0.06- 0.53)	0.1239
Wheeze							
	Week 4	76	0.00±0.46	37	0.00±0.67	0.08 (-0.11- 0.26)	0.4187
	Week 12	73	-0.10±0.58	34	0.00±0.85	0.01 (-0.23- 0.25)	0.9323
	Week 20	72	0.07±0.48	34	-0.06±0.69	0.20 (0.00- 0.39)	0.0471

	Week 24	72	0.01±0.49	33	-0.03±0.64	0.10 (-0.09- 0.28)	0.3031
Chest pain							
	Week 4	76	-0.01±0.58	37	-0.03±0.50	0.00 (-0.20- 0.21)	0.9673
	Week 12	73	0.07±0.56	34	0.06±0.60	-0.01 (-0.21- 0.20)	0.9543
	Week 20	72	0.11±0.46	34	0.03±0.58	0.05 (-0.11- 0.22)	0.5324
	Week 24	72	0.06±0.53	33	-0.09±0.58	0.12 (-0.06- 0.31)	0.1909
Breathlessness on talking							
	Week 4	76	0.03±0.33	37	0.08±0.55	0.02 (-0.13- 0.18)	0.7797
	Week 12	73	0.01±0.46	34	0.15±0.70	-0.02 (-0.22- 0.19)	0.8720
	Week 20	72	-0.06±0.50	34	0.06±0.65	0.02 (-0.19- 0.23)	0.8430
	Week 24	72	-0.06±0.65	33	0.03±0.53	0.06 (-0.17- 0.29)	0.5969
Nocturnal cough							
	Week 4	76	0.03±0.59	37	-0.14±0.54	0.19 (-0.01- 0.39)	0.0568
	Week 12	73	0.10±0.50	34	0.03±0.58	0.09 (-0.09- 0.28)	0.3096
	Week 20	72	0.03±0.58	34	-0.03±0.72	0.09 (-0.15- 0.32)	0.4653
	Week 24	72	-0.01±0.62	33	0.00±0.71	0.01 (-0.23- 0.26)	0.9072

Mean changes from baseline in individual QoL-B-RSS symptom domains between florensocatib and placebo in patient with low symptom burden (QoL-B-RSS≥60).

Abbreviations: QoL-B-RSS=Quality-of-Life Bronchiectasis Respiratory Symptoms Scale; SE: standard error; CI: confidence interval.

Table S6. Mean changes from baseline in individual QoL-B-RSS item scores at day 29 and day 85 in patients receiving inhaled tobramycin versus placebo in the TORNASOL trial. Related to Figure 5.

Items	High-symptom subgroup				Low-symptom subgroup			
	Tobramycin (Mean±SE) n=77	Placebo (Mean±SE) n=63	Mean Difference vs placebo (95%CI)	*P*-value	Tobramycin (Mean±SE) n=89	Placebo (Mean±SE) n=108	Mean Difference vs placebo (95%CI)	*P*-value
Day 29								
Congestion	0.72±0.08	0.15±0.09	0.58 (0.26-0.88)	<0.0001	0.26±0.06	0.05±0.06	0.21 (-0.02-0.43)	0.0894
Daily cough	0.59±0.08	0.11±0.09	0.48 (0.10-0.80)	0.0006	0.10±0.05	-0.12±0.05	0.22 (0.03-0.41)	0.0116
Sputum production	0.55±0.08	0.16±0.09	0.39 (0.08-0.70)	0.0056	0.23±0.06	-0.10±0.05	0.32 (0.11-0.54)	0.0005
Sputum purulence	0.47±0.07	0.09±0.07	0.38 (0.11-0.64)	0.0017	0.38±0.05	-0.11±0.05	0.48 (0.29-0.68)	<0.0001
Breathlessness on daily activity	0.35±0.08	0.10±0.09	0.25 (-0.08-0.51)	0.2755	0.13±0.06	-0.07±0.06	0.19 (-0.03-0.42)	0.1373
Wheeze	0.13±0.06	0.02±0.07	0.11 (-0.14-0.36)	>0.9999	-0.03±0.04	-0.00±0.04	-0.03(-0.18-0.12)	>0.9999
Chest pain	0.20±0.06	0.06±0.06	0.13 (-0.09-0.36)	0.7015	0.04±0.03	-0.03±0.03	0.07 (-0.05-0.20)	0.6790
Breathlessness on talking	0.33±0.08	0.05±0.09	0.28 (-0.03-0.59)	0.1108	-0.08±0.06	-0.03±0.05	-0.05(-0.25-0.15)	>0.9999
Nocturnal cough	0.20±0.07	0.03±0.08	0.17 (-0.12-0.45)	0.6834	0.03±0.05	-0.05±0.04	0.08 (-0.09-0.25)	>0.9999
Day 85								
Congestion	0.65±0.08	0.19±0.09	0.47 (0.15-0.79)	0.0010	0.729±0.06	0.05±0.06	0.25 (0.02-0.48)	0.0283
Daily cough	0.54±0.08	0.23±0.09	0.32 (-0.02-0.65)	0.0737	0.14±0.05	-0.07±0.05	0.21 (0.02-0.40)	0.0217
Sputum production	0.58±0.08	0.26±0.09	0.32 (-0.01-0.64)	0.0666	0.26±0.06	-0.03±0.06	0.29 (0.07-0.51)	0.0038
Sputum purulence	0.43±0.07	0.09±0.08	0.34 (0.05-0.62)	0.0106	0.44±0.06	-0.02±0.05	0.46 (0.26-0.66)	<0.0001
Breathlessness on daily activity	0.49±0.09	0.24±0.10	0.25 (-0.10-0.60)	0.3520	0.13±0.06	-0.01±0.06	0.14 (-0.08-0.37)	0.5471
Wheeze	0.11±0.07	0.15±0.07	-0.04 (-0.30-0.22)	>0.9999	-0.04±0.04	0.03±0.04	-0.06 (-0.21-0.09)	>0.9999
Chest pain	0.23±0.06	-0.02±0.07	0.25 (0.01-0.49)	0.0401	0.11±0.04	-0.00±0.03	0.12 (-0.01-0.24)	0.0984
Breathlessness on talking	0.34±0.08	0.06±0.09	0.27 (-0.05-0.59)	0.1478	-0.03±0.06	-0.08±0.05	0.05 (-0.15-0.23)	>0.9999
Nocturnal cough	0.22±0.08	0.28±0.08	-0.06 (-0.35-0.24)	>0.9999	0.09±0.05	0.02±0.04	0.06 (-0.11-0.24)	>0.9999

The mean difference represents the treatment effect between tobramycin and placebo. And the estimates have been adjusted for baseline symptom score. High-symptom: QoL-B-RSS<60; low-symptom: QoL-B-RSS≥60. QoL-B-RSS: Quality-of-Life Bronchiectasis Respiratory Symptoms Scale; SE: standard error; CI: confidence interval.

Table S7. Individual items comprising the Respiratory Symptom Domain of the Quality-of-Life Bronchiectasis (QoL-B) (questions 29 - 37). Related to STAR Methods.

Q29 Have you felt congestion (fullness) in your chest?	1.A lot	2.A moderate moment	3.A little	4.Not at all
Q30 Have you been coughing during the day?	1.A lot	2.A moderate moment	3.A little	4.Not at all
Q31 Have you had to cough up sputum?	1.A lot	2.A moderate moment	3.A little	4.Not at all
Q32 Has your sputum been mostly:	1.Green with traces of blood or brownish-dark	2.Yellowish-green	3.Clear to yellow	4.Clear
Q33 Have you had shortness of breath when being active, such as when doing housework or gardening?	1.Always	2.Often	3.Sometimes	4.Never
Q34 Have you had wheezing?	1.Always	2.Often	3.Sometimes	4.Never
Q35 Have you had chest pain?	1.Always	2.Often	3.Sometimes	4.Never
Q36 Have you had shortness of breath when talking?	1.Always	2.Often	3.Sometimes	4.Never
Q37 Have you woken during the night because you were coughing?	1.Always	2.Often	3.Sometimes	4.Never

Supplementary figures

Figure S1.

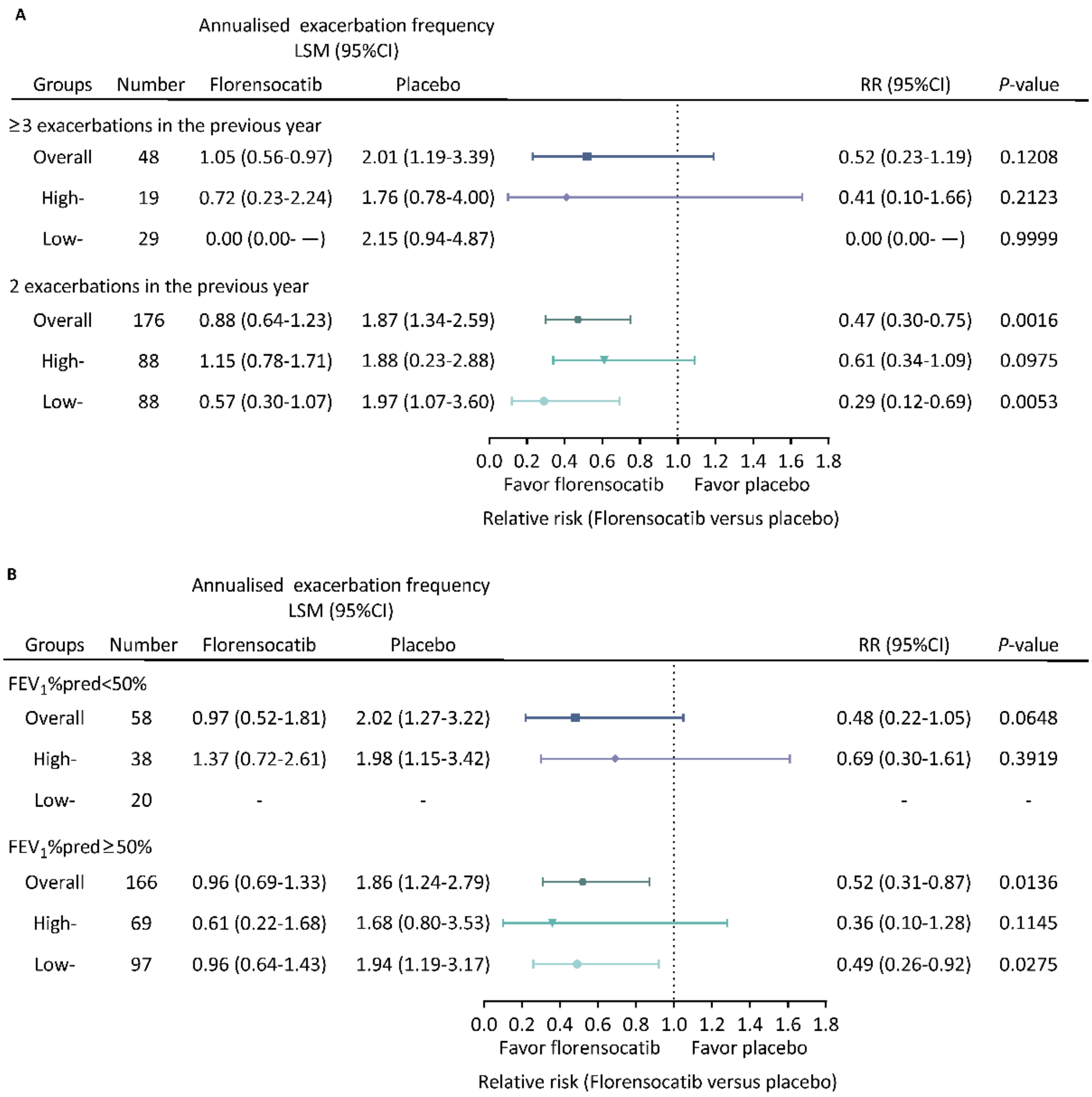

Figure S1. Efficacy of florensocatib in reducing annualised exacerbation rate stratified by symptom burden across disease severity strata in SAVA-BE trial. Related to Figure 1 and Figure 2.

The forest plots show the efficacy of florensocatib versus placebo in reducing annualised frequency of bronchiectasis exacerbation throughout the treatment period of all patients and those with high- or low-symptom burden stratified by different disease severity assessed by exacerbations in the previous one year (≥3 or 2) (A) and FEV1% of predicted (<50% or ≥50%) (B) in the SAVE-BE trial. The annualised exacerbation frequency per person-year is shown as least-squares mean (LSM) with 95% confidence interval (CI). Squares, dots or triangles represent the adjusted mean differences and horizontal lines represent the 95% confidence interval (CI). The relative risk (RR) for the annualised exacerbation frequency is reported for florensocatib versus placebo. Estimates <1 favor florensocatib; >1 favor placebo. Estimates are adjusted for the exacerbation frequency within the previous year (cut-off: 3 events).

High-symptom burden group: QoL-B-RSS <60; low-symptom burden group: QoL-B-RSS ≥60.

Abbreviations: QoL-B-RSS: Quality-of-Life Bronchiectasis Respiratory Symptoms Scale; RR: relative

risk; CI: confidence interval.

Figure S2.

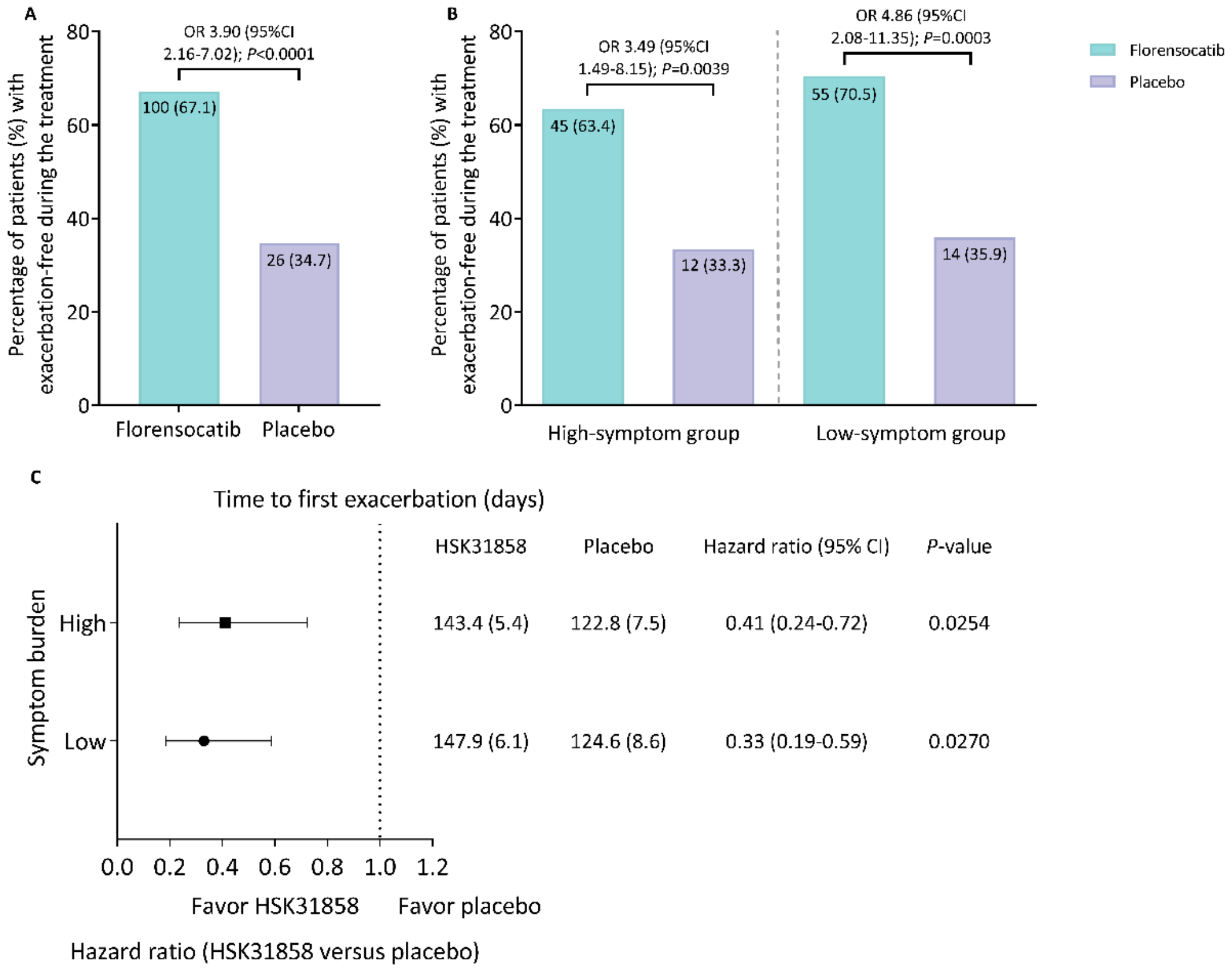

Figure S2. Efficacy of florensocatib in remaining bronchiectasis exacerbation-free and prolonging the time to the first exacerbation, stratified by symptom burden, in the SAVE-BE trial. Related to Figure 1 and Figure 2.

(A) The percentage of patients who remained exacerbation-free during the treatment period according to the SAVE-BE trial group allocation. (B) The percentage of patients who remained exacerbation-free during the treatment period for patients with high-symptom burden and those with low-symptom burden, respectively. The green bars refer to florensocatib, and the purple bars represent placebo. The odd ratio (OR) for remaining exacerbation-free are reported here for florensocatib versus placebo.

(C) The time to the first exacerbation during the treatment period for patients with high- or low-symptom burden, respectively. Time to the first exacerbation (days [standard error]) for each treatment groups in different symptom subgroups over 24 weeks are showed. Squares and dots represent hazard ratio (HR) and horizontal lines represent 95% confidence interval (CI). The HR with 95%CI for the time to the first exacerbation are also reported here for florensocatib versus placebo.

High-symptom burden group: QoL-B-RSS<60; low-symptom burden group: QoL-B-RSS≥60.

Abbreviations: QoL-B-RSS: Quality-of-Life Bronchiectasis Respiratory Symptoms Scale; OR: odd ratio; CI: confidence interval.

Figure S3.

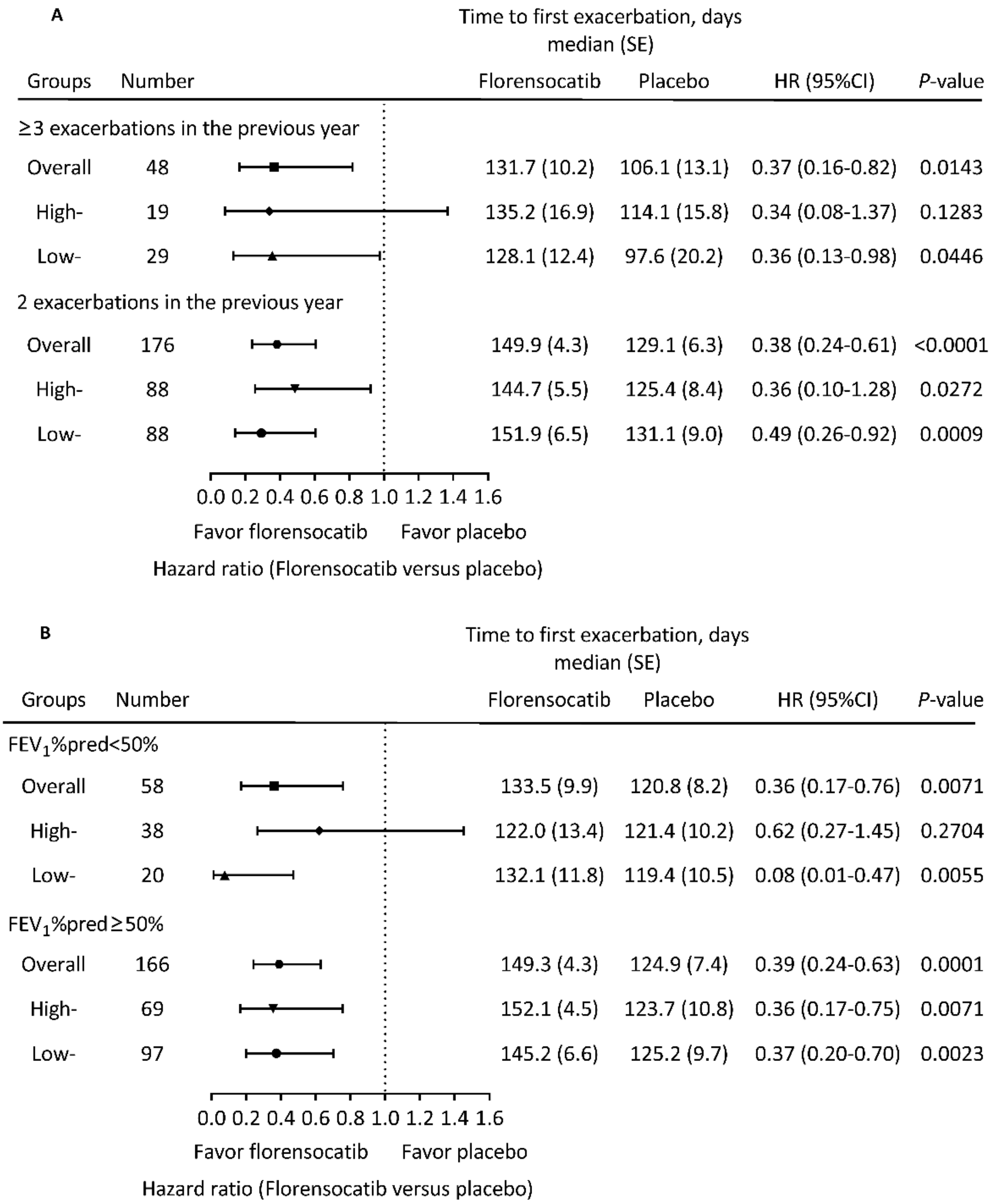

A

Groups	Number	Time to first exacerbation, days median (SE): Florensocatib	Placebo	HR (95%CI)	*P*-value
≥3 exacerbations in the previous year					
Overall	48	131.7 (10.2)	106.1 (13.1)	0.37 (0.16-0.82)	0.0143
High-	19	135.2 (16.9)	114.1 (15.8)	0.34 (0.08-1.37)	0.1283
Low-	29	128.1 (12.4)	97.6 (20.2)	0.36 (0.13-0.98)	0.0446
2 exacerbations in the previous year					
Overall	176	149.9 (4.3)	129.1 (6.3)	0.38 (0.24-0.61)	<0.0001
High-	88	144.7 (5.5)	125.4 (8.4)	0.36 (0.10-1.28)	0.0272
Low-	88	151.9 (6.5)	131.1 (9.0)	0.49 (0.26-0.92)	0.0009

B

Groups	Number	Time to first exacerbation, days median (SE): Florensocatib	Placebo	HR (95%CI)	*P*-value
FEV_1%pred<50%					
Overall	58	133.5 (9.9)	120.8 (8.2)	0.36 (0.17-0.76)	0.0071
High-	38	122.0 (13.4)	121.4 (10.2)	0.62 (0.27-1.45)	0.2704
Low-	20	132.1 (11.8)	119.4 (10.5)	0.08 (0.01-0.47)	0.0055
FEV_1%pred≥50%					
Overall	166	149.3 (4.3)	124.9 (7.4)	0.39 (0.24-0.63)	0.0001
High-	69	152.1 (4.5)	123.7 (10.8)	0.36 (0.17-0.75)	0.0071
Low-	97	145.2 (6.6)	125.2 (9.7)	0.37 (0.20-0.70)	0.0023

Figure S3. Efficacy of florensocatib in prolonging the time to the first exacerbation stratified by symptom burden across disease severity strata in SAVA-BE trial. Related to Figure 1 and Figure 2.

The time to the first exacerbation during the treatment period for all patients and those with high- or low-symptom burden stratified by different disease severity assessed by exacerbations in the previous year (≥3 or 2) (A) and FEV1% predicted (<50% or ≥50%) (B) in the SAVE-BE trial. Time to the first exacerbation (median days [standard error]) for each treatment groups in different subgroups over 24 weeks are showed. Squares, dots or triangles represent hazard ratio (HR) and horizontal lines represent 95% confidence interval (CI). The HR with 95%CI for the time to the first exacerbation are also reported here for florensocatib versus placebo.

High-symptom burden group: QoL-B-RSS<60; low-symptom burden group: QoL-B-RSS≥60.

Abbreviations: QoL-B-RSS: Quality-of-Life Bronchiectasis Respiratory Symptoms Scale; HR: hazard ratio; CI: confidence interval.

Figure S4.

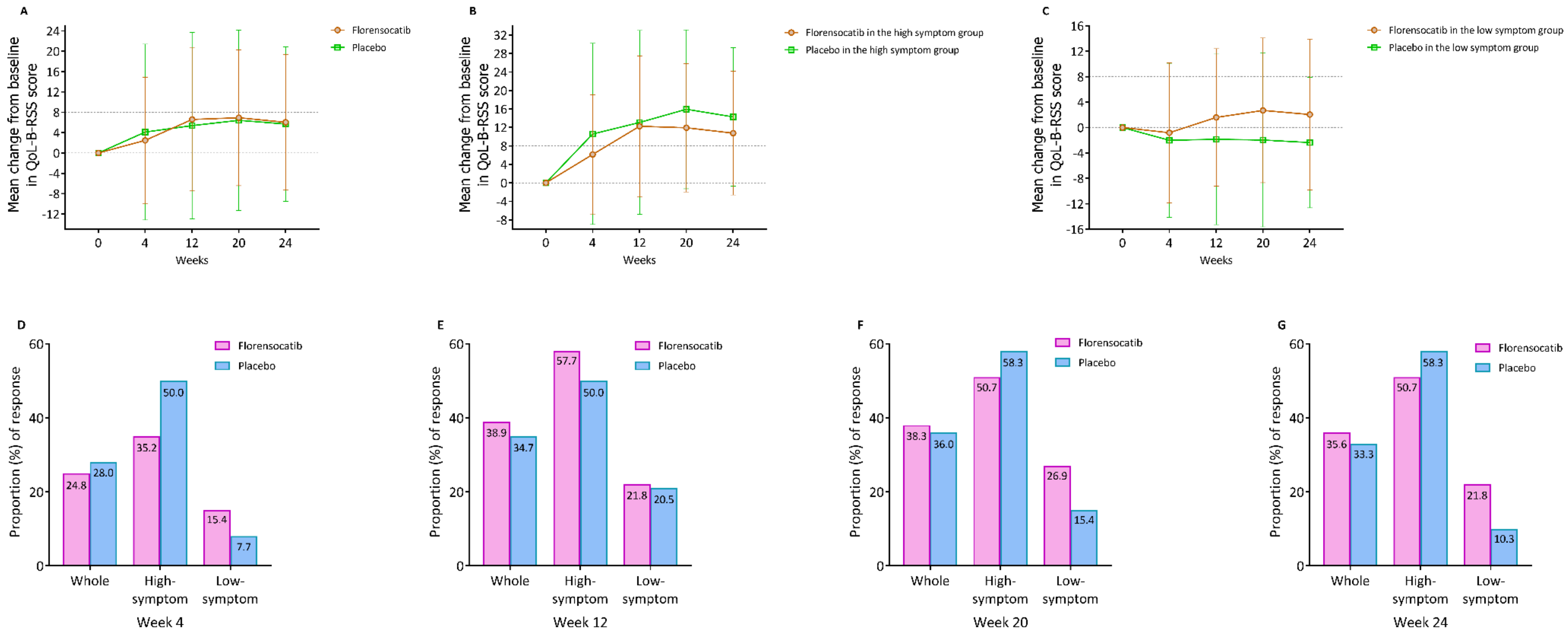

Figure S4. Change in QoL-B-RSS from baseline and the proportion of patients achieving a clinically meaningful improvement in QoL-B-RSS in the SAVE-BE trial. Related to Figure 1 and Figure 2.

(A-C) Mean change from baseline for QoL-B-RSS in whole patients and patients stratified by baseline symptom burden assessed by QoL-B-RSS and treatment arm. Panel A represents the whole patients in the SAVE-BE trial. Panel B stands for those with high symptom burden and Panel C indicates those with low symptom burden. Time point: Week 0 (baseline), 4, 12, 20 and 24.

(D-G) Bar charts display the percentage of patients with improvement (increase) from baseline in QoL-B-RSS score of ≥8 points (established MCID threshold for the

respiratory symptoms domain) at week 4, 12, 20 and 24, respectively. All *P*>0.05 for comparison between florensocatib and placebo.
High symptom burden: <60; low symptom burden: ≥60. Abbreviations: QoL-B-RSS=Quality-of-Life Bronchiectasis Respiratory Symptom Scale. MCID: minimun clinically important difference.

Figure S5.

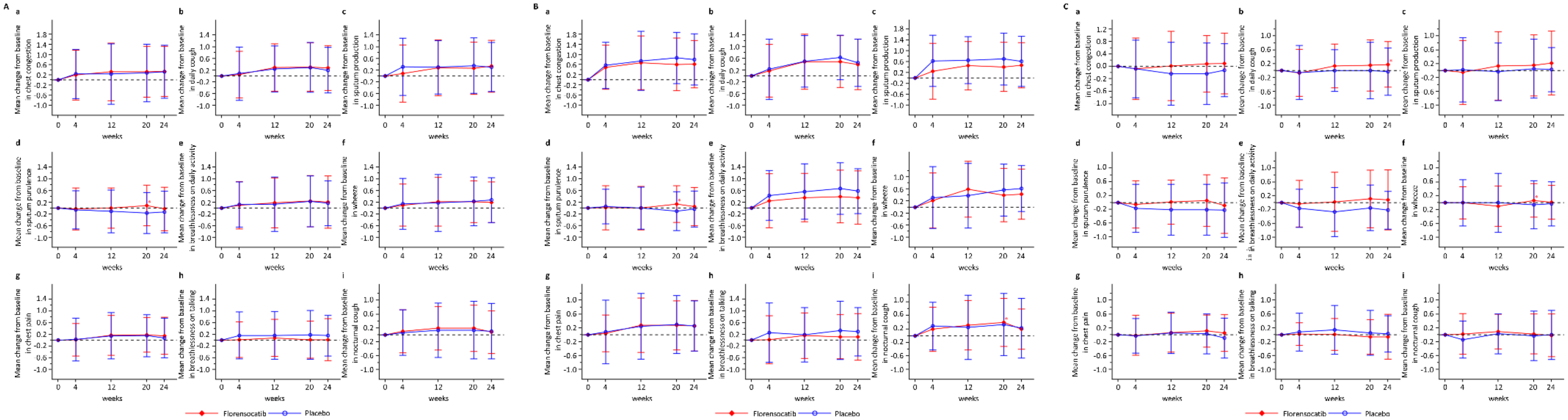

Figure S5. Changes from baseline in individual respiratory symptoms of QoL-B-RSS in patients receiving florensocatib versus placebo in the SAVE-BE trial. Related to Figure 1 and Figure 2.

(A) Mean changes from baseline in individual QoL-B-RSS symptom domains are presented for the florensocatib group (red line) and the placebo group (blue line) in patients receiving florensocatib versus placebo.

(B) Mean changes from baseline in individual QoL-B-RSS symptom domains are presented for the florensocatib group (red line) and the placebo group (blue line) in patients with high-symptom burden receiving florensocatib versus placebo.

(C) Mean changes from baseline in individual QoL-B-RSS symptom domains are presented for the florensocatib group (red line) and the placebo group (blue line) in patients with low-symptom burden receiving florensocatib versus placebo.

Panels show the following domains: a) chest congestion; b) daily cough; c) sputum production; d) sputum purulence; e) breathlessness on daily activity; f) wheeze; g) chest pain; h) breathlessness on talking; and i) nocturnal cough. Abbreviations: QoL-B-RSS=Quality-of-Life Bronchiectasis Respiratory Symptoms Scale.

Figure S6.

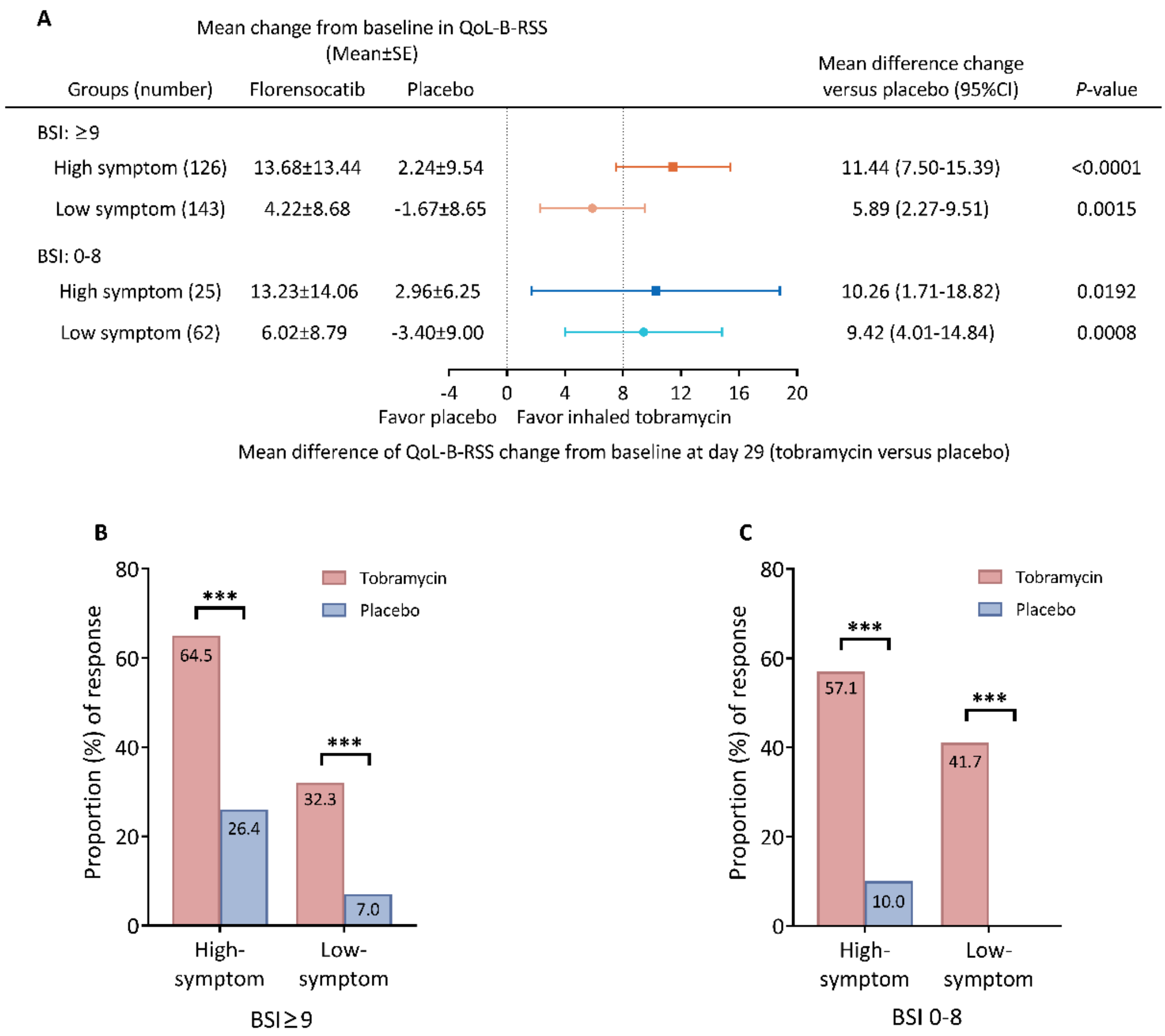

Figure S6. Change in QoL-B-RSS from baseline and the proportion of patients achieving a clinically meaningful improvement in QoL-B-RSS in patients with different symptom burden stratified by disease severity in TORNASOL trial. Related to Figure 3 and Figure 4.

(A) Mean change from baseline for QoL-B-RSS of all patients and those with high- or low-symptom burden stratified by different disease severity assessed by bronchiectasis severity index (BSI) and treatment arm at day 29 (the primary outcome time point). (B-C) Bart charts display the percentage of patients with an improvement (increase) from baseline in QoL-B-RSS score of ≥8 points for those with BSI ≥9 and BSI 0-8, respectively, at day 29. ***: *P*<0.0001.

Severe disease: BSI≥9; Moderate and mild disease: BSI 0-8; High-symptom burden: <60; low-symptom burden: ≥60. Abbreviations: QoL-B-RSS=Quality-of-Life Bronchiectasis Respiratory Symptoms Scale. MCID: minimum clinically important difference.

Figure S7.

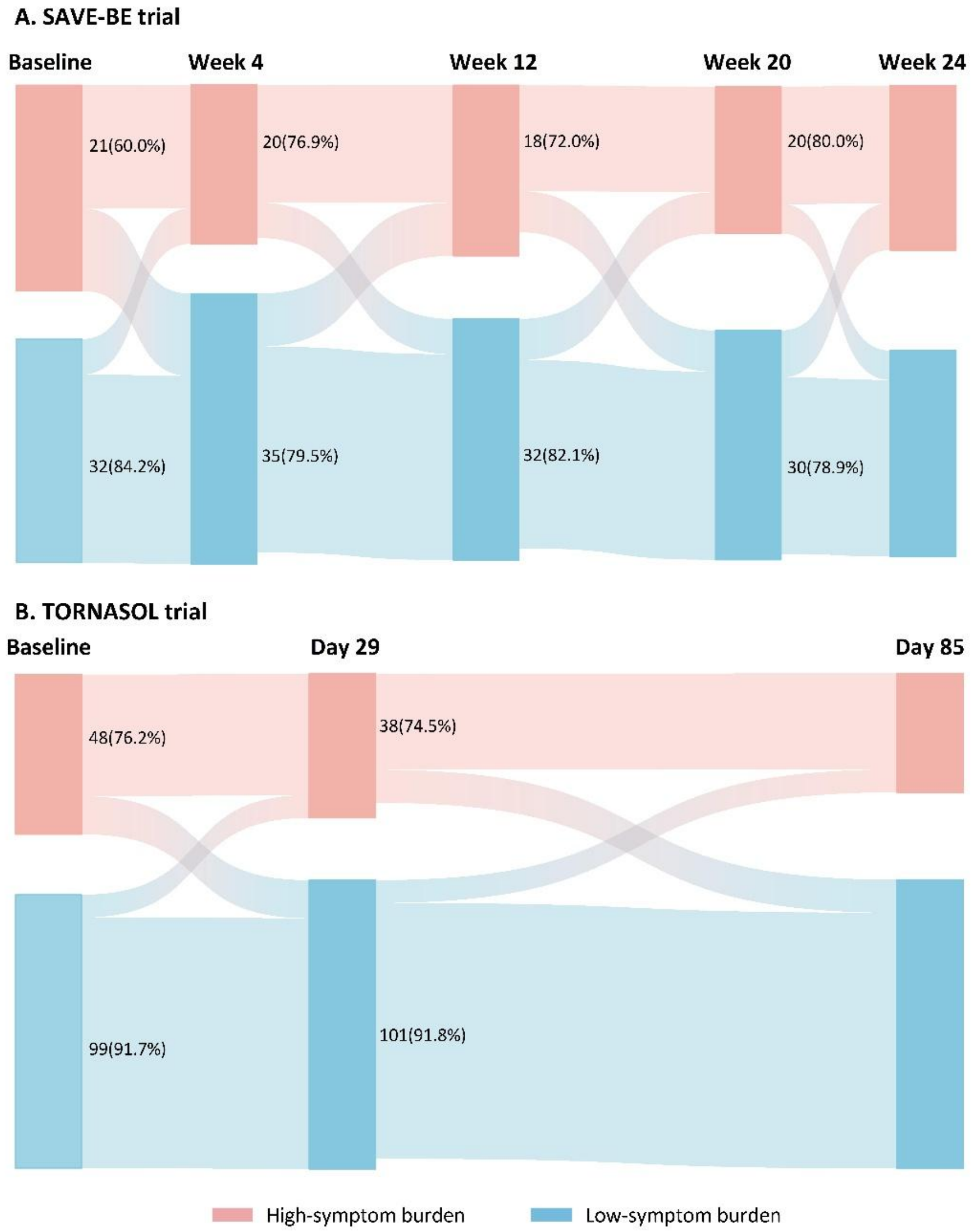

Figure S7. Change of symptom burden subgroups in placebo arm in SAVE-BE trial and TORNASOL trial. Related to Figure 1 and Figure 2. Related to Figure 2 and Figure 4.

Sankey diagram illustrating the transitions in symptom burden subgroups during the trial period for patients with bronchiectasis in placebo arm in (A) SAVE-BE trial and (B) TORNASOL trials. Vertical nodes represent the distribution of patients across two symptom burden subgroups at each study visit: high- (QoL-B-RSS<60, red) and low- (QoL-B-RSS≥60, blue) symptom burden. The width of connecting flows is proportional to the number of patients transitioning between subgroups or remaining in the same subgroup from one time point to the next. Numerical labels on the flows indicate the absolute number and percentages of patients for each persistence.
